# Supplementary material for: An evaluation of bird and bat mortality at wind turbines in the Northeastern United States
Source: PLoS One. 2020 Aug 28;15(8):e0238034. doi: 10.1371/journal.pone.0238034 (PMC7454995; doi:10.1371/journal.pone.0238034)
Supplement: S1 Appendix — Data from reports submitted to the US Fish and Wildlife Service via either a special purpose utility or scientific collecting permit between 2008 and 2017. (DOCX) [file pone.0238034.s001.docx]

**S1 Appendix**. **Bird species and total fatalities from 44 wind projects in the Northeastern US.** Data from reports submitted to the US Fish and Wildlife Service via either a special purpose utility or scientific collecting permit between 2008 and 2017.

| **ORDER, *Family*,** Species | Fatalities | **ORDER, *Family*,** Species | Fatalities |
| --- | --- | --- | --- |
| **ACCIPITRIFORMES** | **53** | ***Icteriidae*** | ***1*** |
| ***Accipitridae*** | ***53*** | Yellow-breasted Chat | 1 |
| Bald Eagle | 1 | ***Mimidae*** | ***14*** |
| Broad-winged Hawk | 5 | Brown Thrasher | 5 |
| Northern Goshawk | 1 | Gray Catbird | 9 |
| Red-tailed Hawk | 34 | ***Paridae*** | ***1*** |
| Sharp-shinned Hawk | 10 | Black-capped Chickadee | 1 |
| Unidentified Hawk | 2 | ***Parulidae*** | ***576*** |
| **ANSERIFORMES** | **25** | American Redstart | 31 |
| ***Anatidae*** | ***25*** | Bay-breasted Warbler | 6 |
| American Black Duck | 1 | Black-and-white Warbler | 26 |
| Canada Goose | 2 | Blackburnian Warbler | 22 |
| Gadwall | 2 | Blackpoll Warbler | 28 |
| Hooded Merganser | 2 | Black-throated Blue Warbler | 59 |
| Mallard | 3 | Black-throated Green Warbler | 21 |
| Redhead | 1 | Blue-winged Warbler | 2 |
| Ring-necked Duck | 1 | Canada Warbler | 4 |
| Ruddy Duck | 2 | Cape May Warbler | 4 |
| Unidentified Waterfowl | 2 | Chestnut-sided Warbler | 22 |
| Wood Duck | 9 | Common Yellowthroat | 49 |
| **APODIFORMES** | **26** | Connecticut Warbler | 2 |
| ***Apodidae*** | ***12*** | Hooded Warbler | 5 |
| Chimney Swift | 12 | Kentucky Warbler | 1 |
| ***Trochilidae*** | ***14*** | Magnolia Warbler | 99 |
| Ruby-throated Hummingbird | 14 | Mourning Warbler | 3 |
| **CAPRIMULGIFORMES** | **5** | Nashville Warbler | 3 |
| ***Caprimulgidae*** | ***5*** | Northern Parula | 25 |
| Common Nighthawk | 4 | Northern Waterthrush | 6 |
| Eastern Whip-poor-will | 1 | Ovenbird | 33 |
| **CATHARTIFORMES** | **42** | Palm Warbler | 2 |
| ***Cathartidae*** | ***42*** | Pine Warbler | 8 |
| Black Vulture | 2 | Prairie Warbler | 1 |
| Turkey Vulture | 40 | Tennessee Warbler | 11 |
| **CHARADRIIFORMES** | **36** | Unidentified Warbler | 46 |
| ***Charadriidae*** | ***5*** | Wilson's Warbler | 2 |
| Killdeer | 5 | Yellow Warbler | 4 |
| ***Laridae*** | ***19*** | Yellow-rumped Warbler | 51 |
| Ring-billed Gull | 12 | Yellow-throated Warbler | 2 |
| Unidentified Gull | 7 | ***Passerellidae*** | ***1*** |
| ***Scolopacidae*** | ***12*** | Savannah Sparrow | 1 |
| American Woodcock | 11 | ***Passeridae*** | ***3*** |
| Semipalmated Sandpiper | 1 | House Sparrow | 3 |
| **COLUMBIFORMES** | **13** | ***Regulidae*** | ***145*** |
| ***Columbidae*** | ***13*** | Golden-crowned Kinglet | 122 |
| Mourning Dove | 5 | Ruby-crowned Kinglet | 21 |
| Rock Pigeon | 8 | Unidentified Kinglet | 2 |
| **CORACIIFORMES** | **2** | ***Sittidae*** | ***18*** |
| ***Alcedinidae*** | ***2*** | Red-breasted Nuthatch | 18 |
| Belted Kingfisher | 2 | ***Sturnidae*** | ***13*** |
| **CUCULIFORMES** | **41** | European Starling | 13 |
| ***Cuculidae*** | ***41*** | ***Troglodytidae*** | ***6*** |
| Black-billed Cuckoo | 19 | Winter Wren | 6 |
| Yellow-billed Cuckoo | 22 | ***Turdidae*** | ***74*** |
| **FALCONIFORMES** | **2** | American Robin | 10 |
| ***Falconidae*** | ***2*** | Gray-cheeked Thrush | 3 |
| American Kestrel | 2 | Hermit Thrush | 11 |
| **GALLIFORMES** | **75** | Swainson's Thrush | 18 |
| ***Phasianidae*** | ***75*** | Unidentified Thrush | 11 |
| Ruffed Grouse | 55 | Veery | 7 |
| Wild Turkey | 20 | Wood Thrush | 14 |
| **GRUIFORMES** | **4** | ***Tyrannidae*** | ***33*** |
| ***Rallidae*** | ***4*** | Acadian Flycatcher | 2 |
| Sora | 3 | Eastern Kingbird | 1 |
| Virginia Rail | 1 | Eastern Phoebe | 2 |
| **PASSERIFORMES** | **1592** | Eastern Wood-Pewee | 4 |
| ***Alaudidae*** | ***1*** | Great Crested Flycatcher | 1 |
| Horned Lark | 1 | Least Flycatcher | 2 |
| ***Bombycillidae*** | ***19*** | Unidentified Flycatcher | 8 |
| Cedar Waxwing | 19 | Yellow-bellied Flycatcher | 13 |
| ***Cardinalidae*** | ***12*** | ***Unknown Family*** | ***116*** |
| Indigo Bunting | 4 | Unidentified Passerine | 5 |
| Rose-breasted Grosbeak | 4 | Unidentified Songbird | 102 |
| Scarlet Tanager | 4 | Unidentified Sparrow | 9 |
| ***Certhiidae*** | ***3*** | ***Vireonidae*** | ***440*** |
| Brown Creeper | 3 | Blue-headed Vireo | 33 |
| ***Corvidae*** | ***19*** | Philadelphia Vireo | 9 |
| American Crow | 8 | Red-eyed Vireo | 376 |
| Blue Jay | 3 | Unidentified Vireo | 17 |
| Common Raven | 4 | Warbling Vireo | 1 |
| Unidentified Corvid | 4 | White-eyed Vireo | 3 |
| ***Emberizidae*** | ***68*** | Yellow-throated Vireo | 1 |
| Chipping Sparrow | 7 | **PELECANIFORMES** | **2** |
| Dark-eyed Junco | 16 | ***Ardeidae*** | ***2*** |
| Eastern Towhee | 7 | Great Blue Heron | 1 |
| Field Sparrow | 3 | Unidentified Bittern | 1 |
| Fox Sparrow | 2 | **PICIFORMES** | **31** |
| Lincoln's Sparrow | 3 | ***Picidae*** | ***31*** |
| Song Sparrow | 10 | Downy Woodpecker | 3 |
| White-throated Sparrow | 20 | Northern Flicker | 4 |
| ***Fringillidae*** | ***3*** | Unidentified Woodpecker | 4 |
| American Goldfinch | 1 | Yellow-bellied Sapsucker | 20 |
| Pine Siskin | 1 | **PODICIPEDIFORMES** | **1** |
| Purple Finch | 1 | ***Podicipedidae*** | ***1*** |
| ***Hirundinidae*** | ***12*** | Pied-billed Grebe | 1 |
| Barn Swallow | 1 | **STRIGIFORMES** | **2** |
| Cliff Swallow | 2 | ***Strigidae*** | ***2*** |
| Tree Swallow | 9 | Barred Owl | 1 |
| ***Icteridae*** | ***14*** | Northern Saw-whet Owl | 1 |
| Baltimore Oriole | 1 | **UNKNOWN ORDER** | **87** |
| Bobolink | 3 | ***Unknown Family*** | ***87*** |
| Brown-headed Cowbird | 2 | Unidentified Bird | 86 |
| Common Grackle | 1 | Unidentified Raptor | 1 |
| Red-winged Blackbird | 6 | **Grand Total** | **2039** |
| Unidentified Blackbird | 1 |  |  |
