# Supplementary figures and images for: An evaluation of bird and bat mortality at wind turbines in the Northeastern United States
Source: PLoS One. 2020 Aug 28;15(8):e0238034. doi: 10.1371/journal.pone.0238034 (PMC7454995; doi:10.1371/journal.pone.0238034)

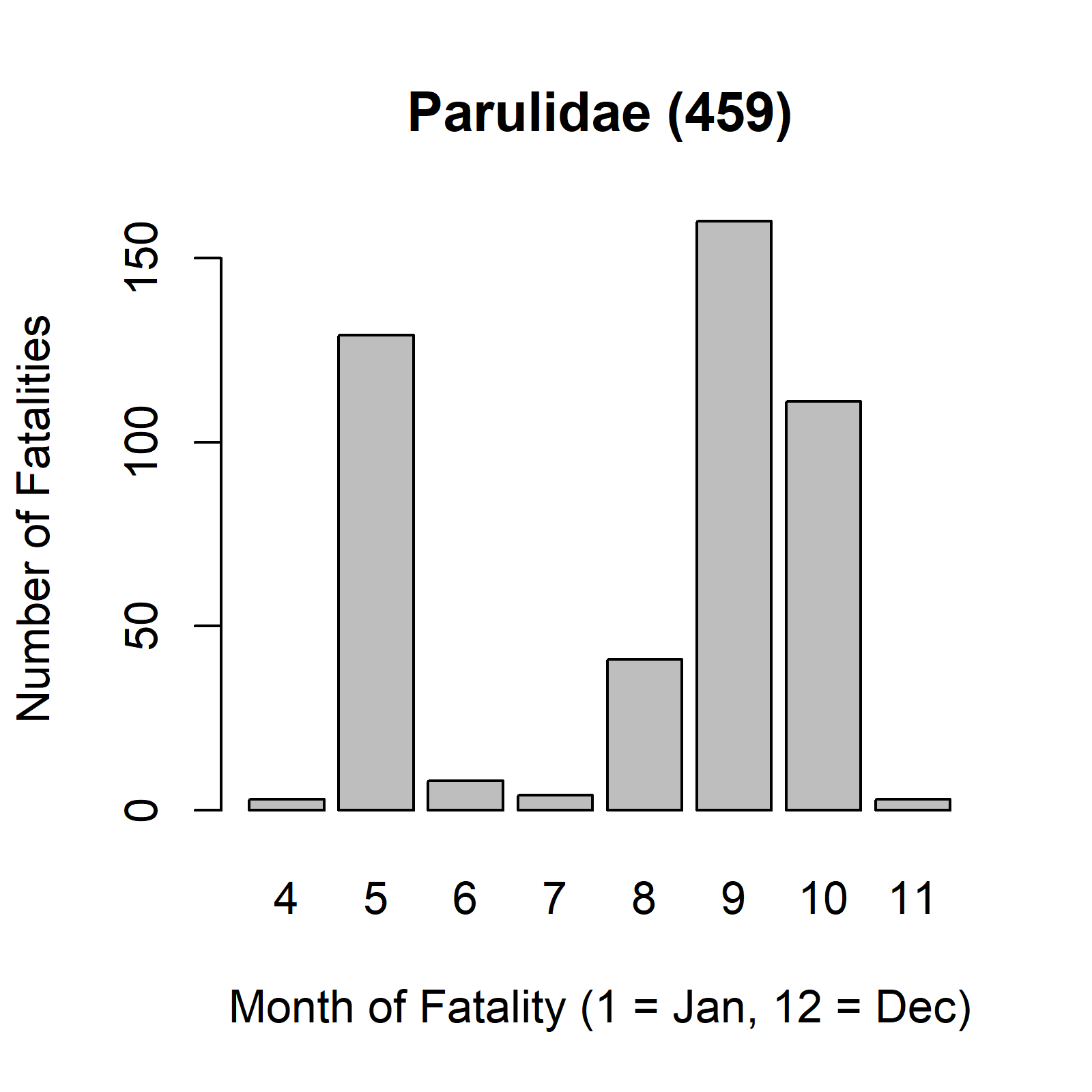

Supplement: S1 Fig — Data from reports submitted to the US Fish and Wildlife Service by 44 wind facilities in the Northeastern US. Sample size given in parentheses. (PNG) [file pone.0238034.s002.png]

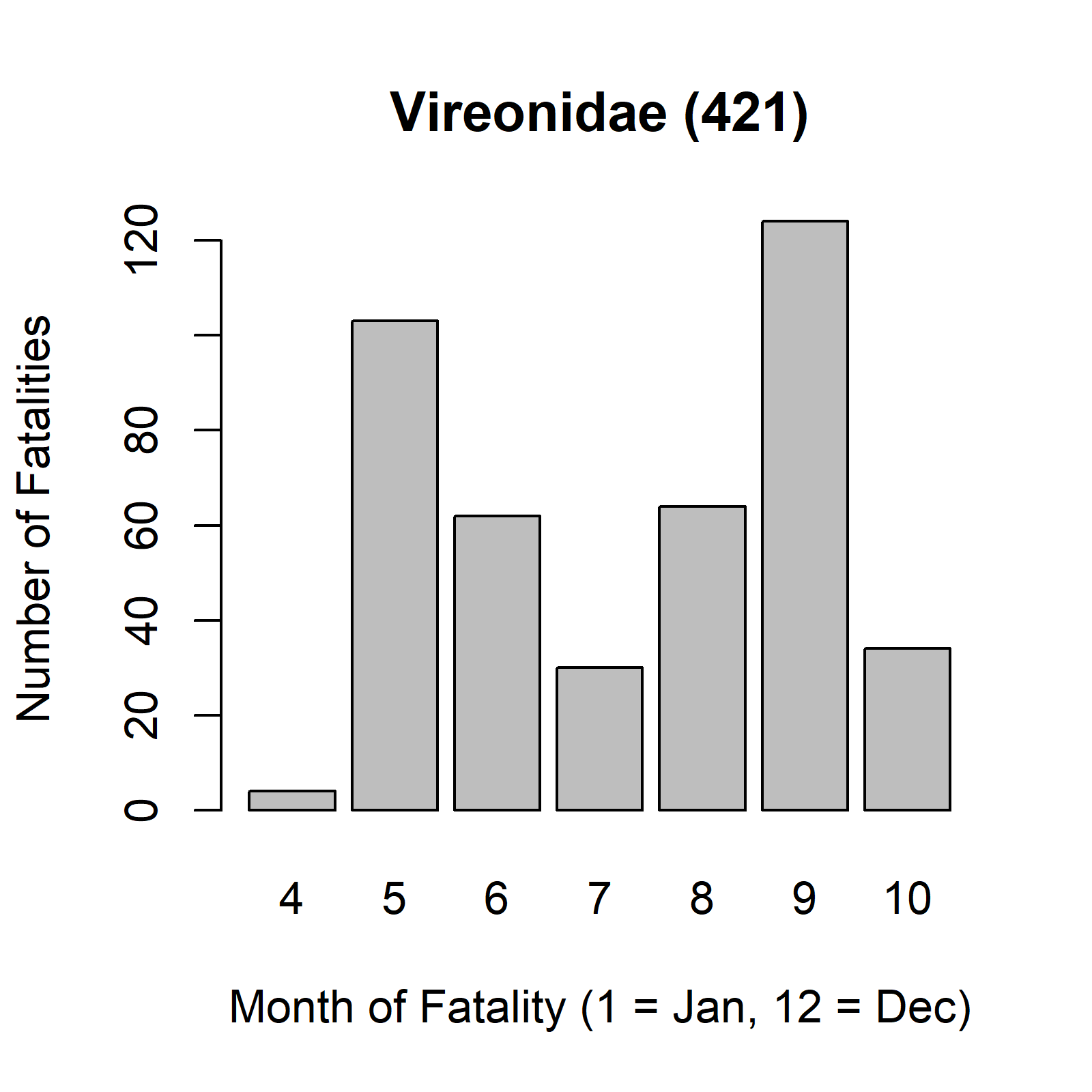

Supplement: S2 Fig — Data from reports submitted to the US Fish and Wildlife Service by 44 wind facilities in the Northeastern US. Sample size given in parentheses. (PNG) [file pone.0238034.s003.png]

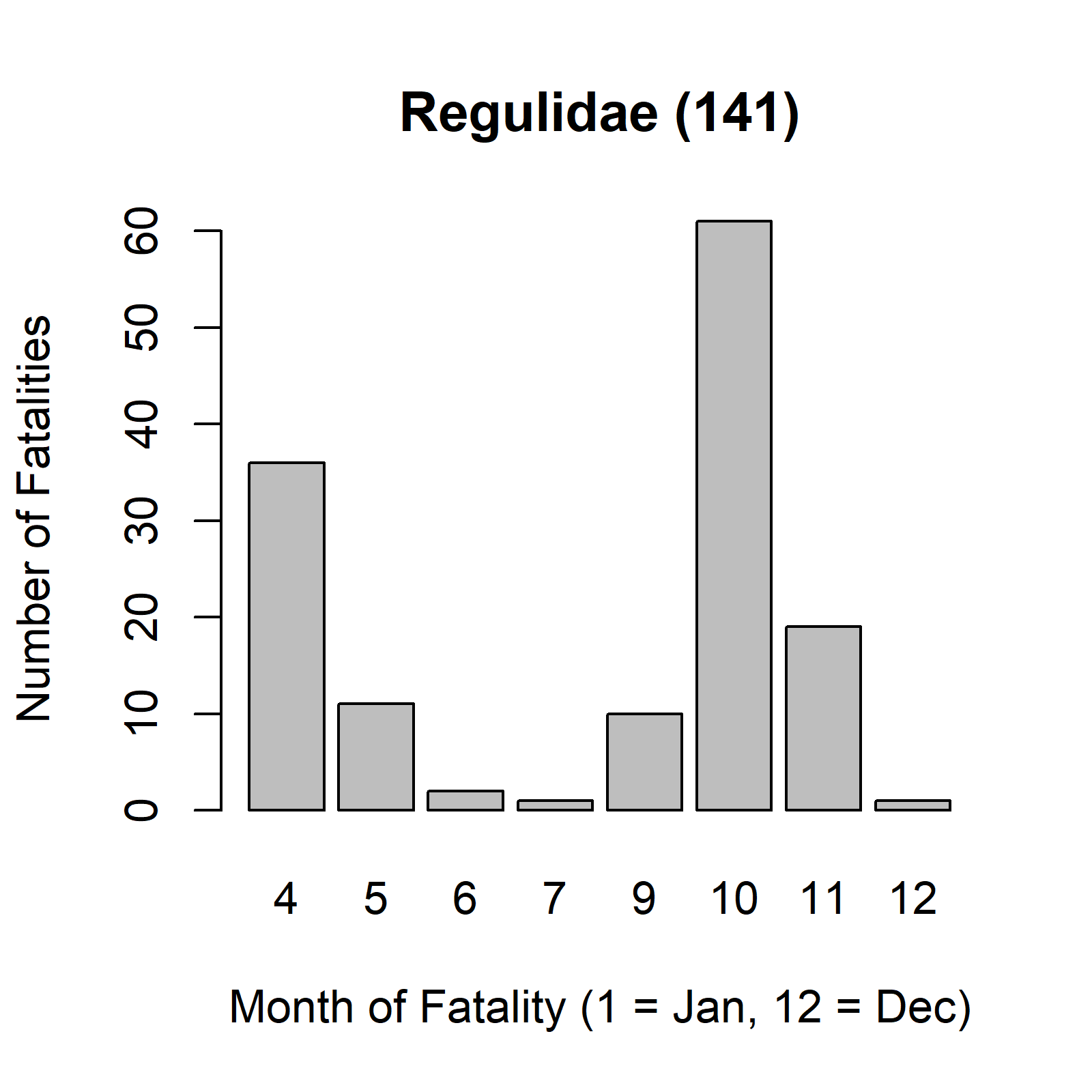

Supplement: S3 Fig — Data from reports submitted to the US Fish and Wildlife Service by 44 wind facilities in the Northeastern US. Sample size given in parentheses. (PNG) [file pone.0238034.s004.png]

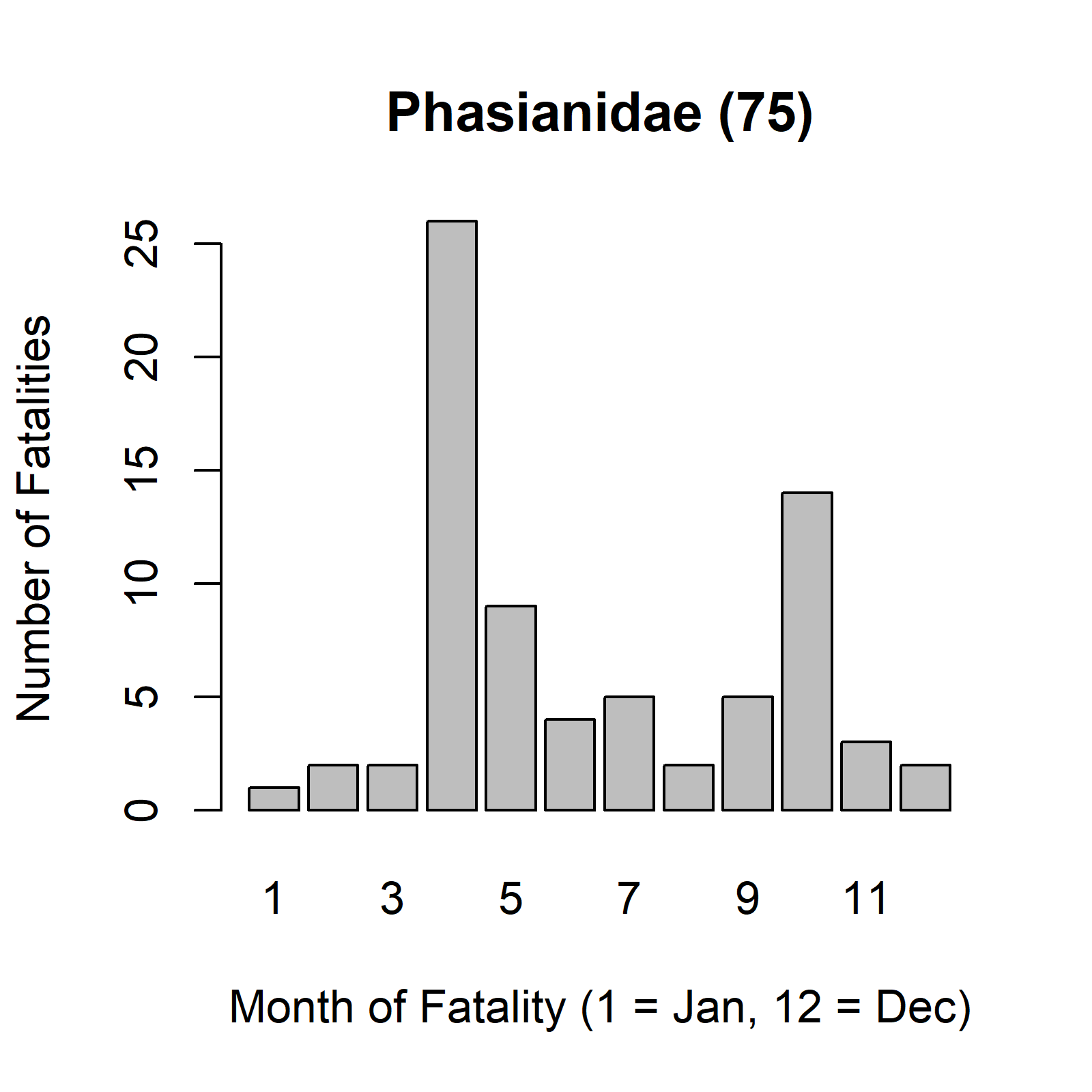

Supplement: S4 Fig — Data from reports submitted to the US Fish and Wildlife Service by 44 wind facilities in the Northeastern US. Sample size given in parentheses. (PNG) [file pone.0238034.s005.png]

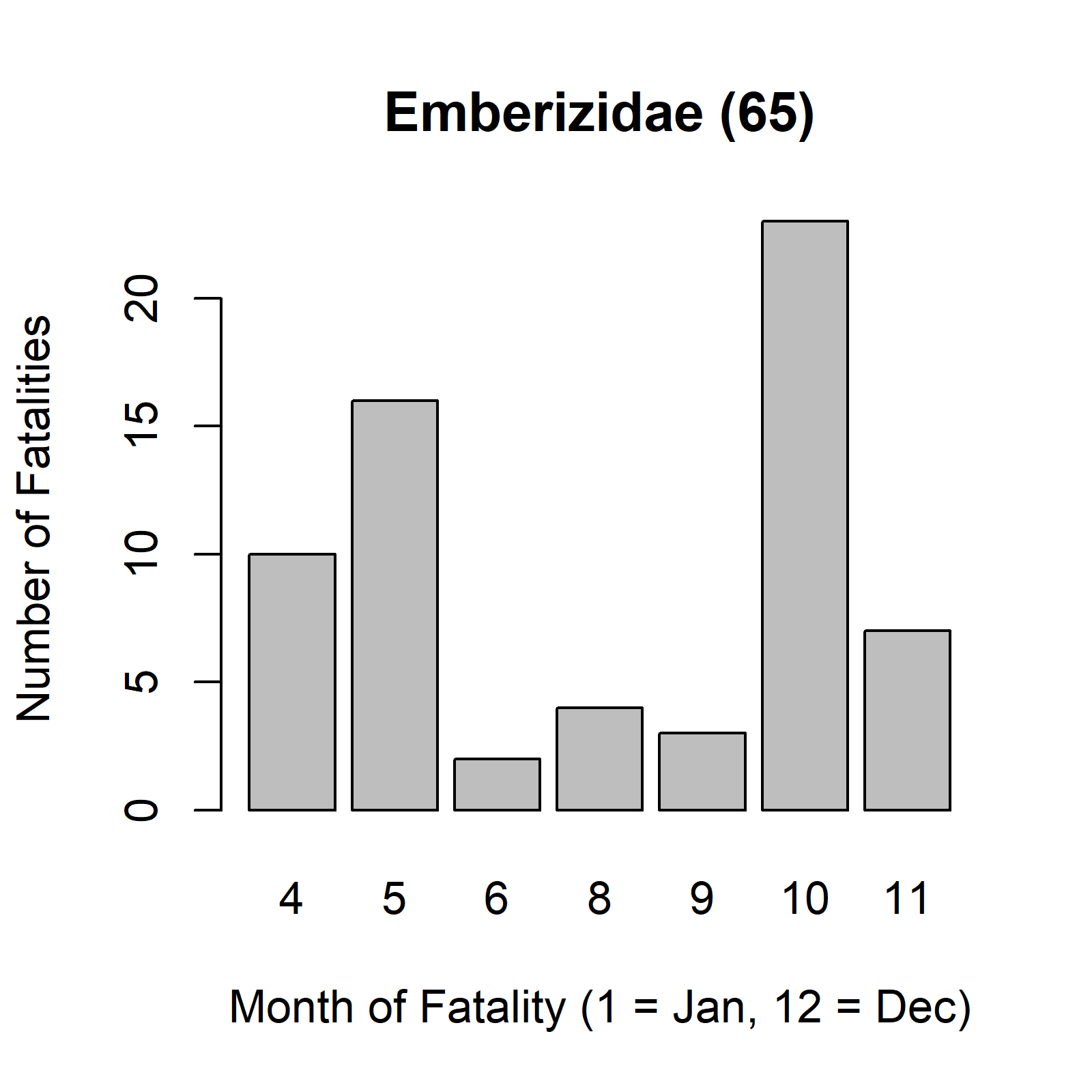

Supplement: S5 Fig — Data from reports submitted to the US Fish and Wildlife Service by 44 wind facilities in the Northeastern US. Sample size given in parentheses. (PNG) [file pone.0238034.s006.png]

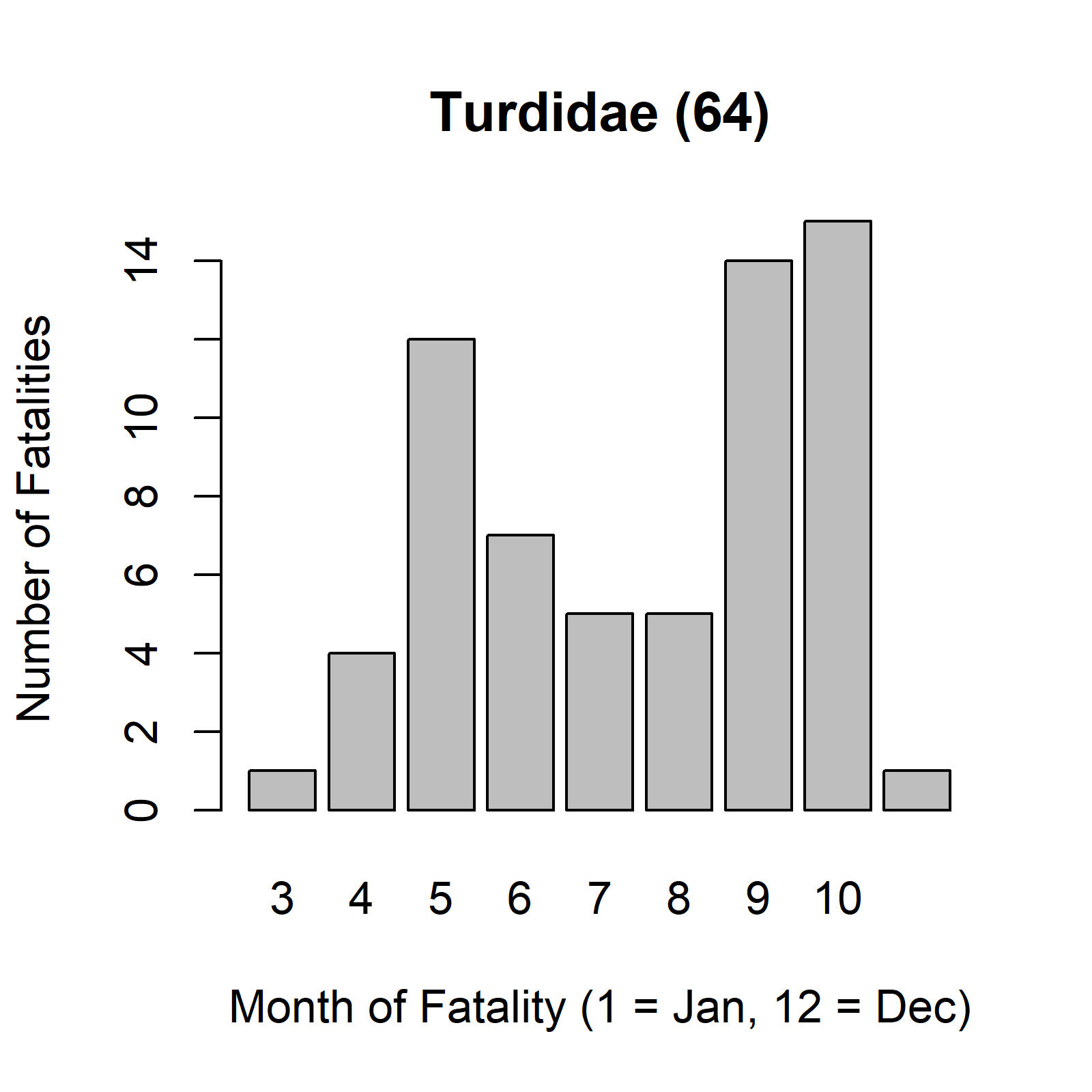

Supplement: S6 Fig — Data from reports submitted to the US Fish and Wildlife Service by 44 wind facilities in the Northeastern US. Sample size given in parentheses. (PNG) [file pone.0238034.s007.png]

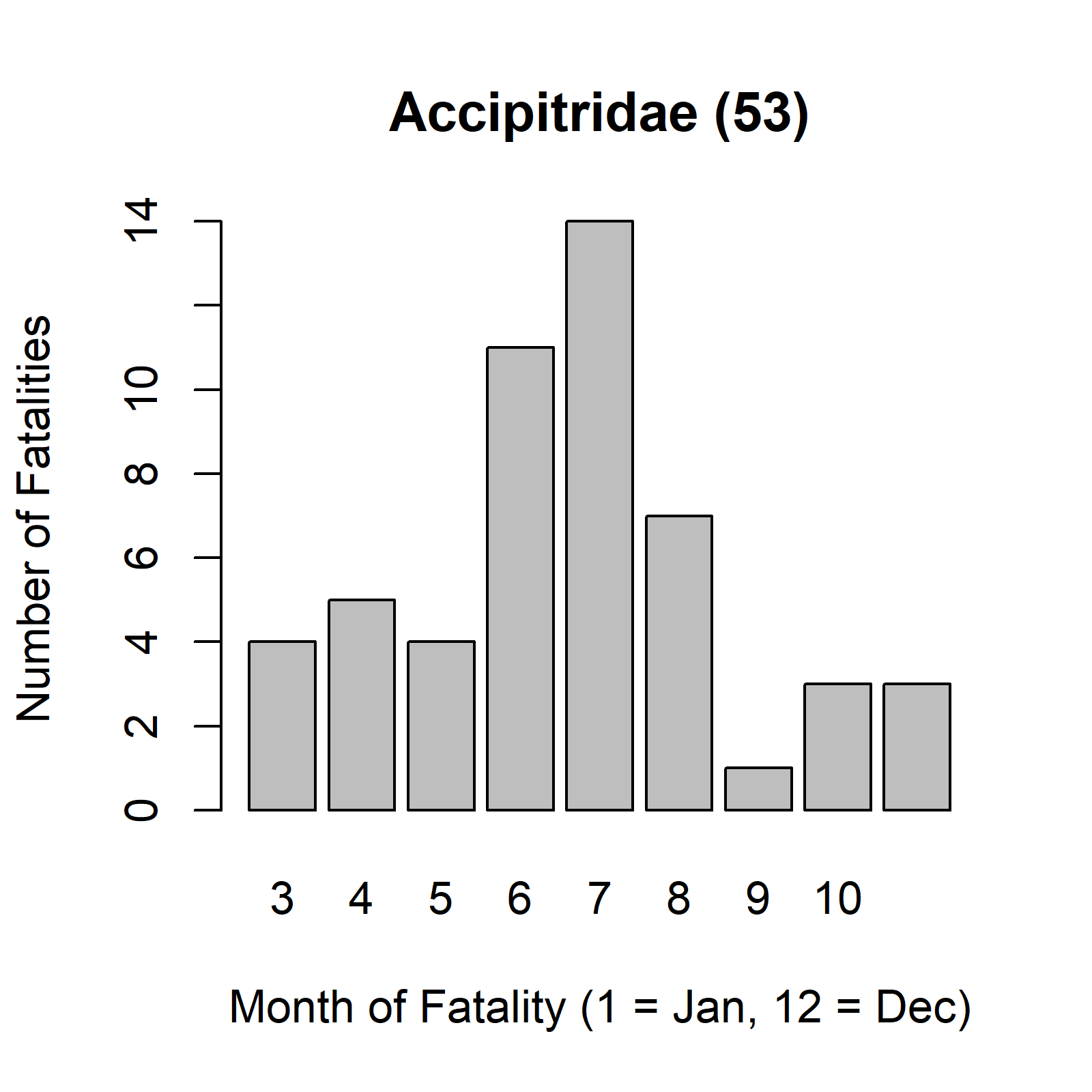

Supplement: S7 Fig — Data from reports submitted to the US Fish and Wildlife Service by 44 wind facilities in the Northeastern US. Sample size given in parentheses. (PNG) [file pone.0238034.s008.png]

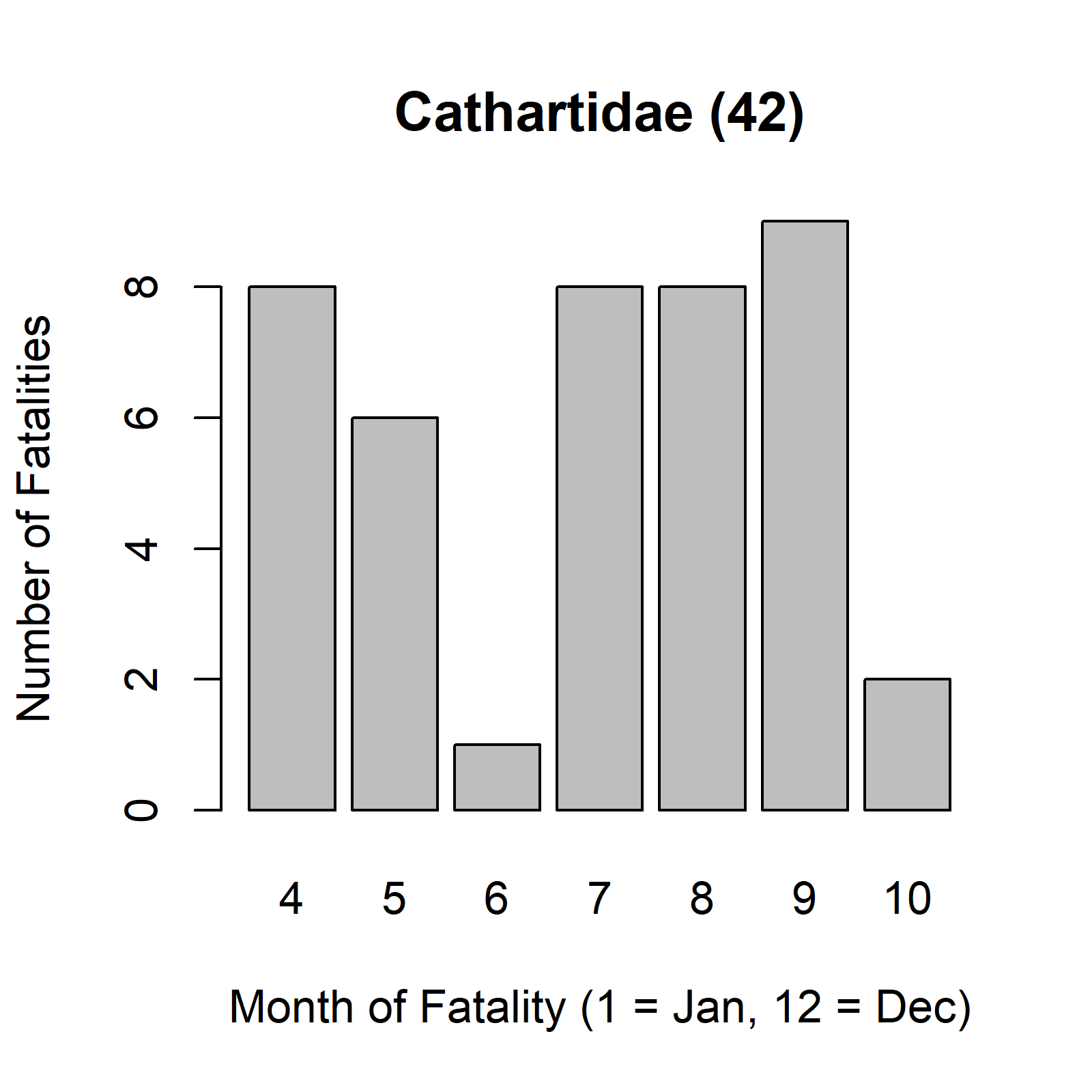

Supplement: S8 Fig — Data from reports submitted to the US Fish and Wildlife Service by 44 wind facilities in the Northeastern US. Sample size given in parentheses. (PNG) [file pone.0238034.s009.png]

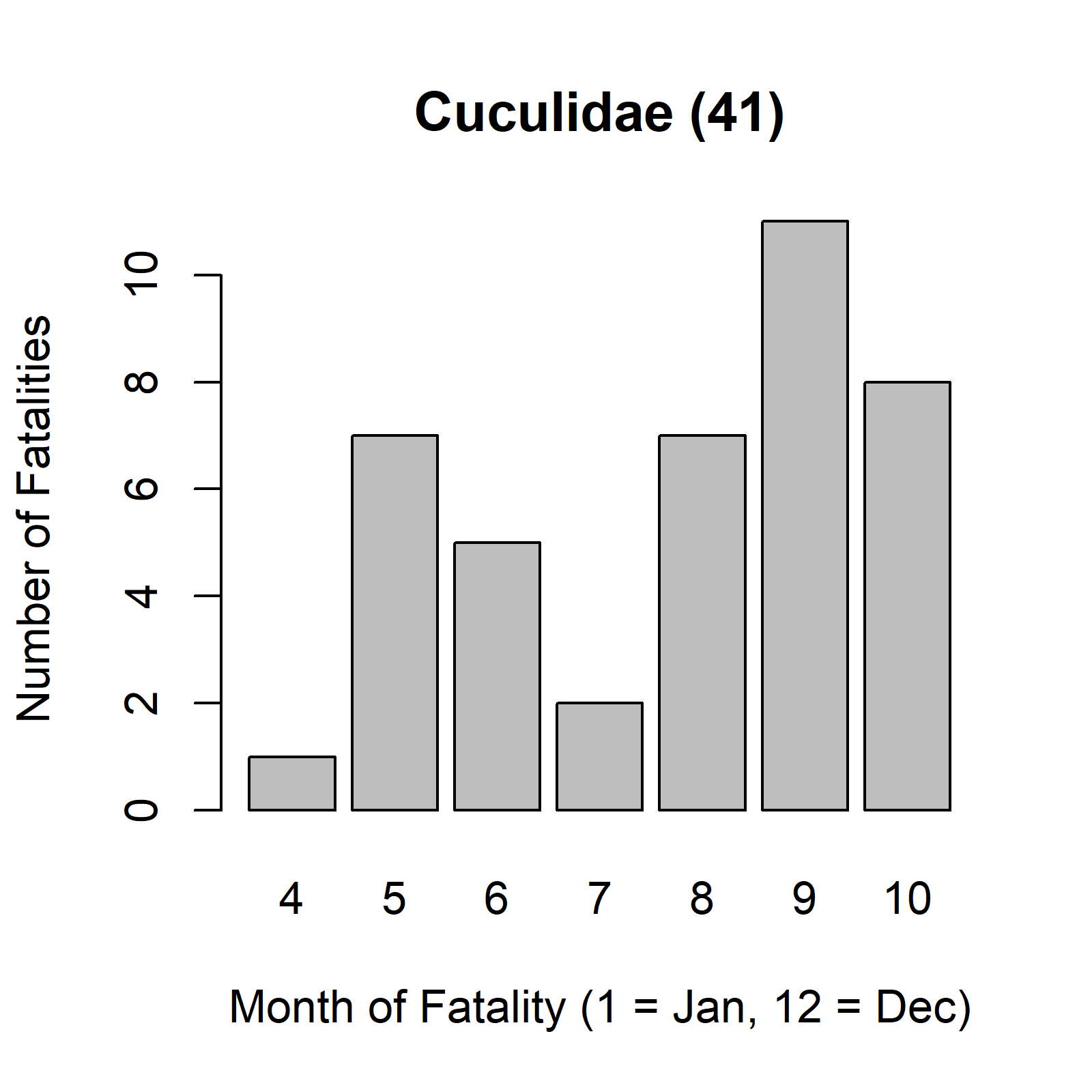

Supplement: S9 Fig — Data from reports submitted to the US Fish and Wildlife Service by 44 wind facilities in the Northeastern US. Sample size given in parentheses. (PNG) [file pone.0238034.s010.png]

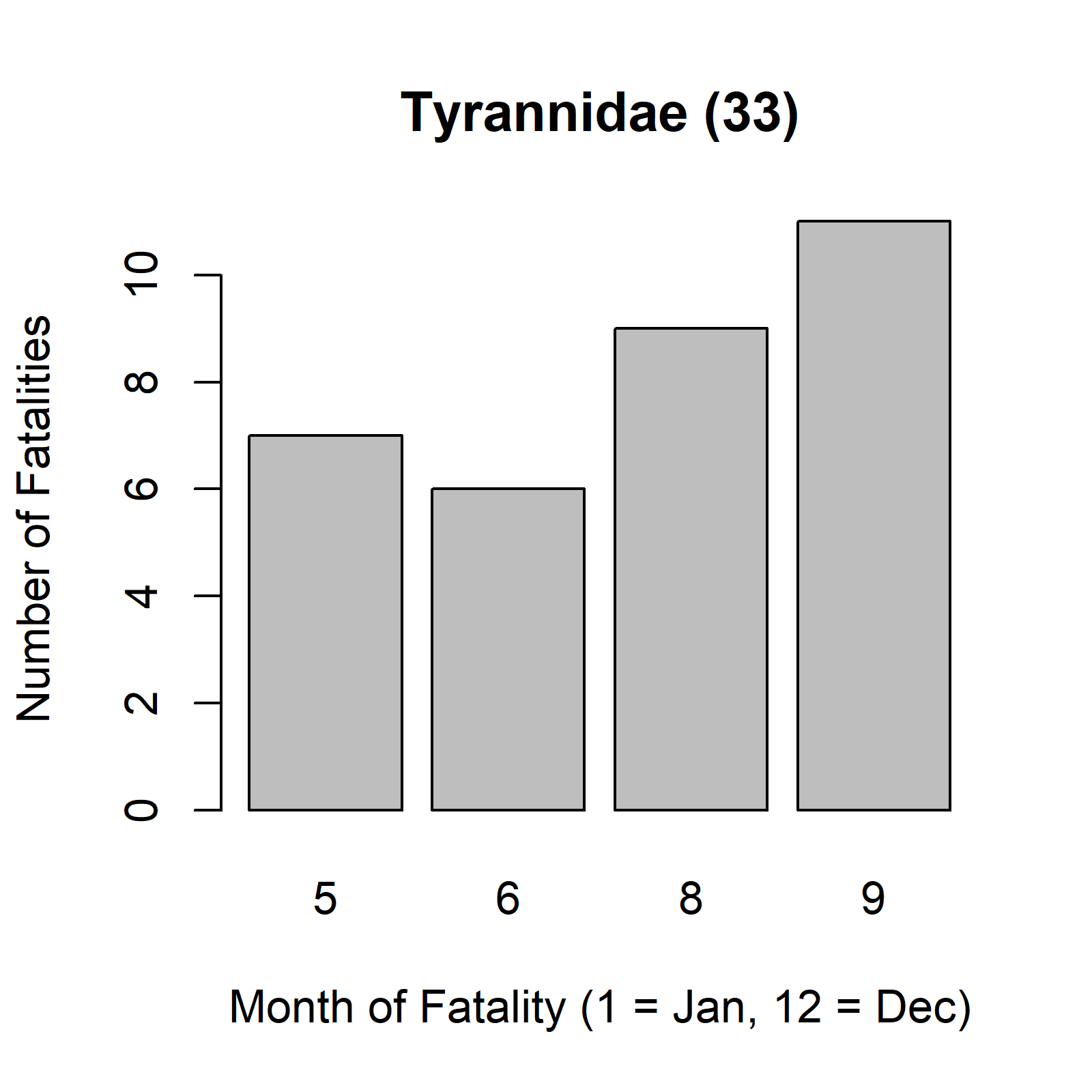

Supplement: S10 Fig — Data from reports submitted to the US Fish and Wildlife Service by 44 wind facilities in the Northeastern US. Sample size given in parentheses. (PNG) [file pone.0238034.s011.png]

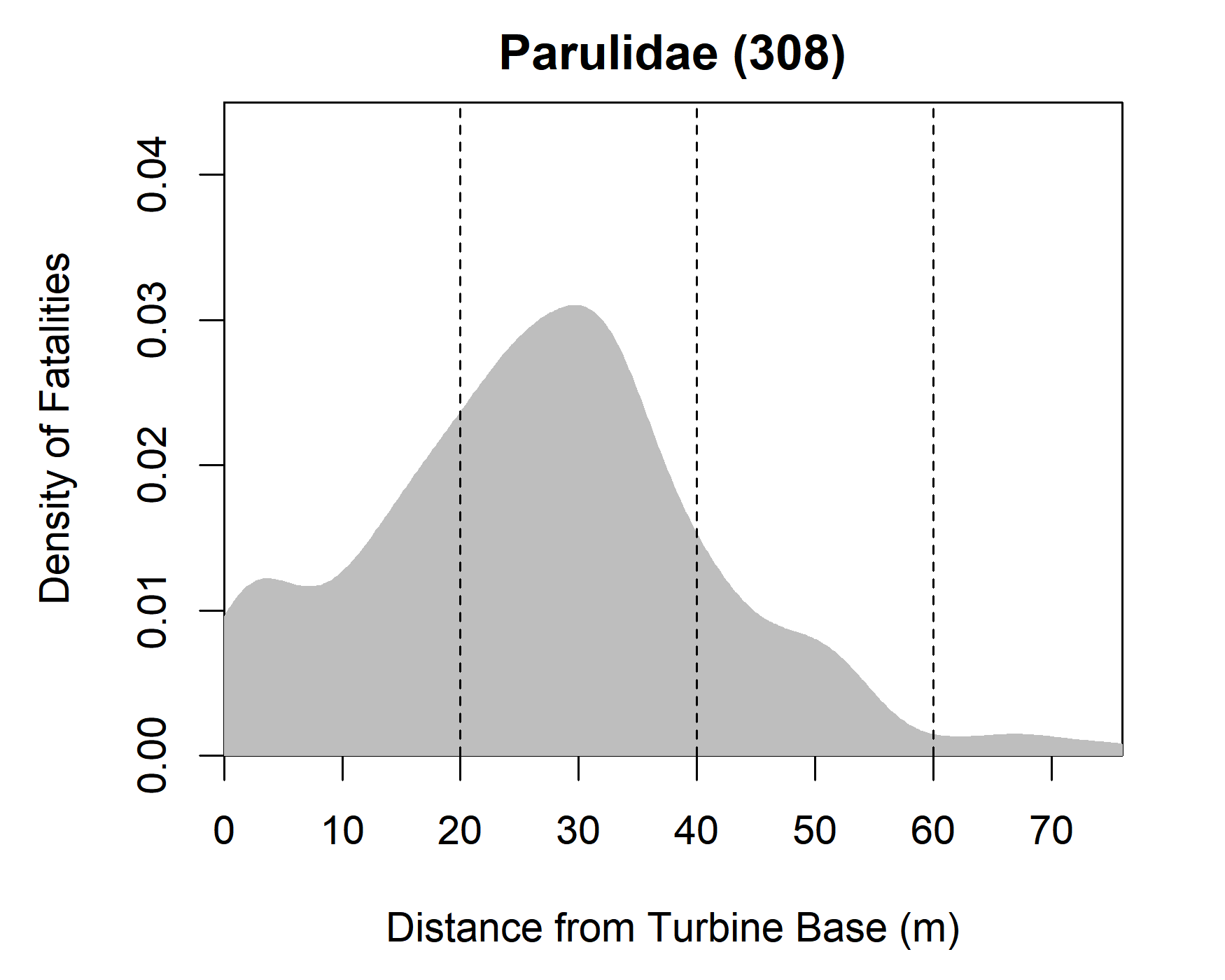

Supplement: S11 Fig — Data from publicly available reports and from reports submitted to the US Fish and Wildlife Service by 44 wind facilities in the Northeastern US. Sample size given in parentheses. (TIFF) [file pone.0238034.s012.tiff]

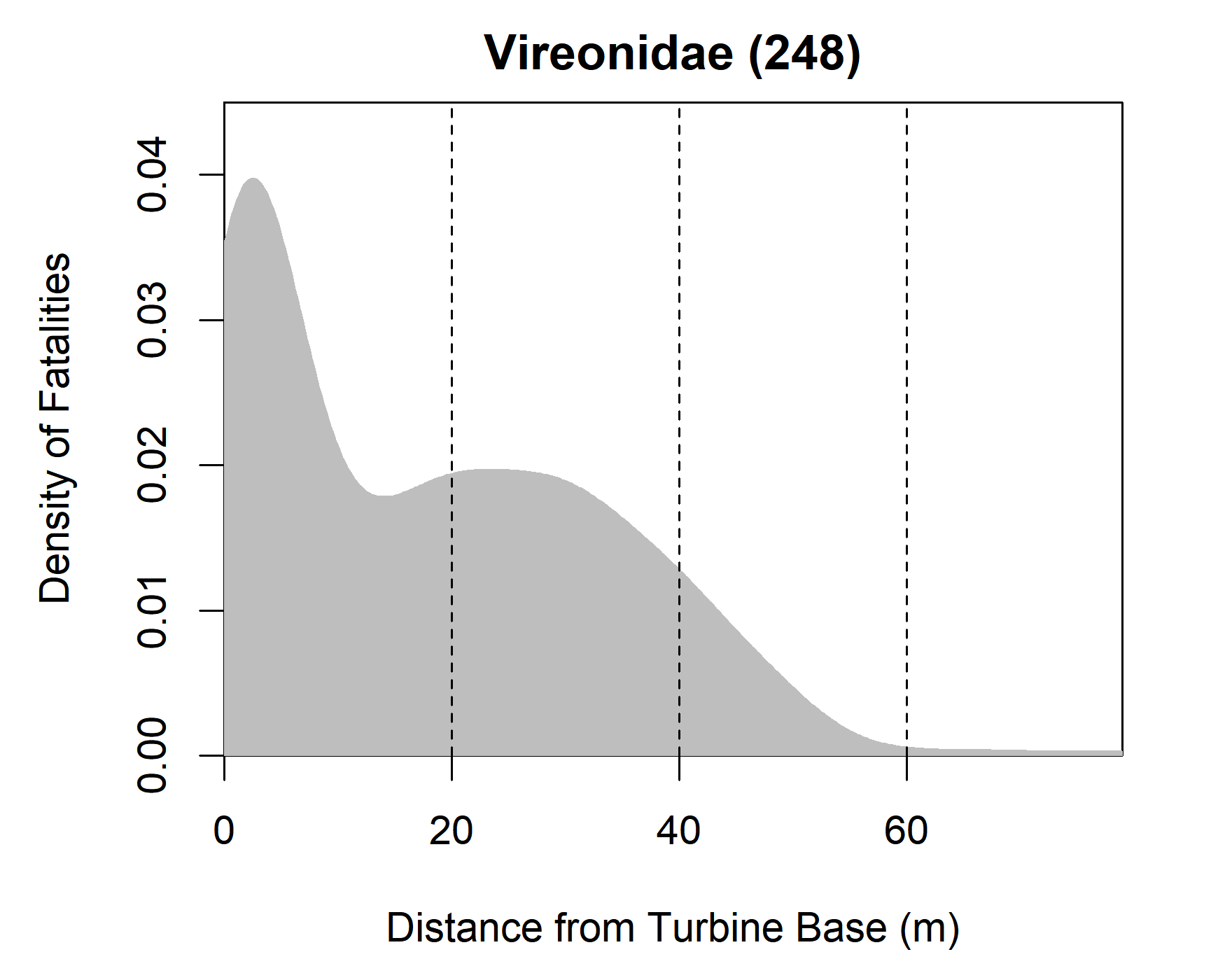

Supplement: S12 Fig — Data from publicly available reports and from reports submitted to the US Fish and Wildlife Service by 44 wind facilities in the Northeastern US. Sample size given in parentheses. (TIFF) [file pone.0238034.s013.tiff]

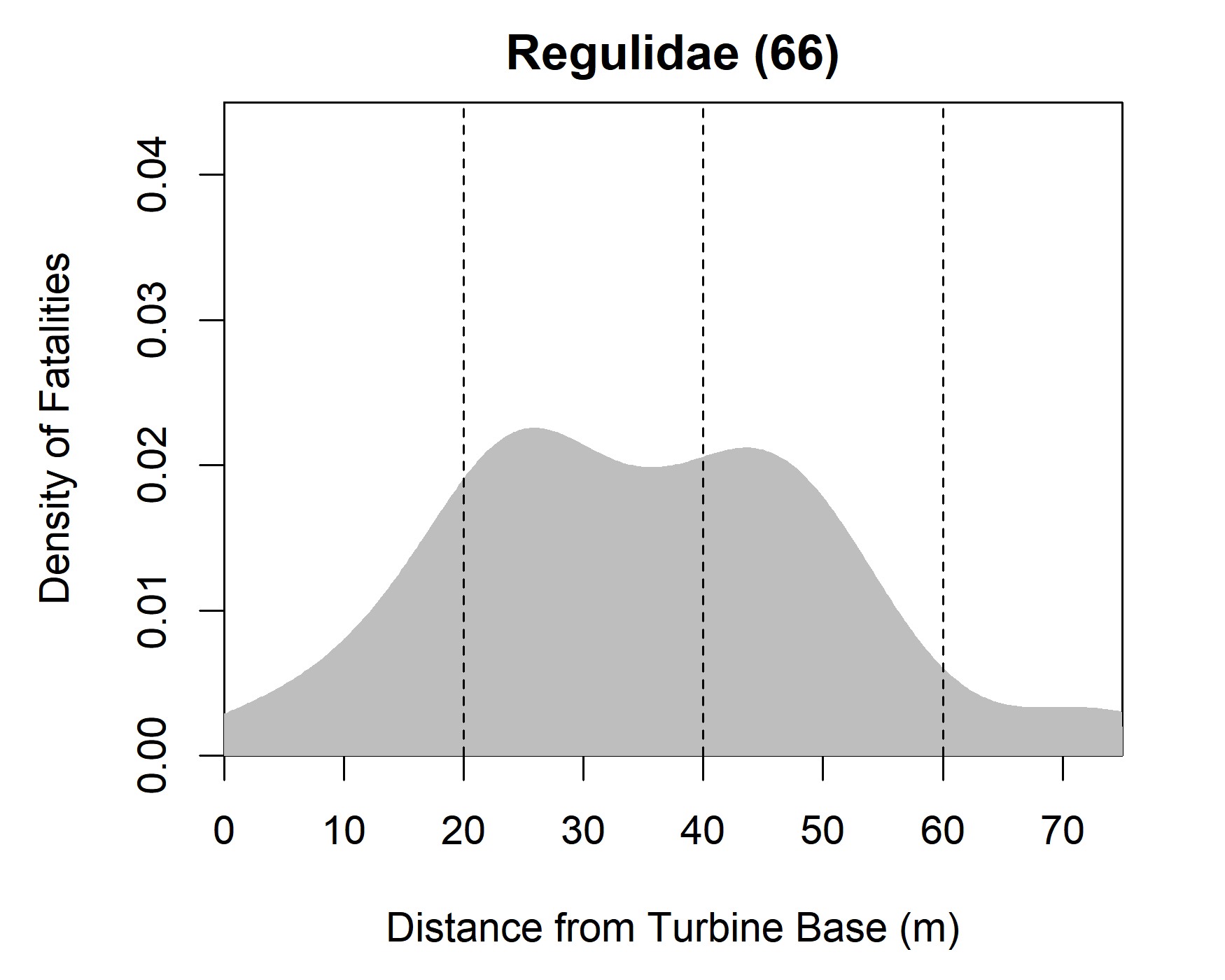

Supplement: S13 Fig — Data from publicly available reports and from reports submitted to the US Fish and Wildlife Service by 44 wind facilities in the Northeastern US. Sample size given in parentheses. (TIFF) [file pone.0238034.s014.tiff]

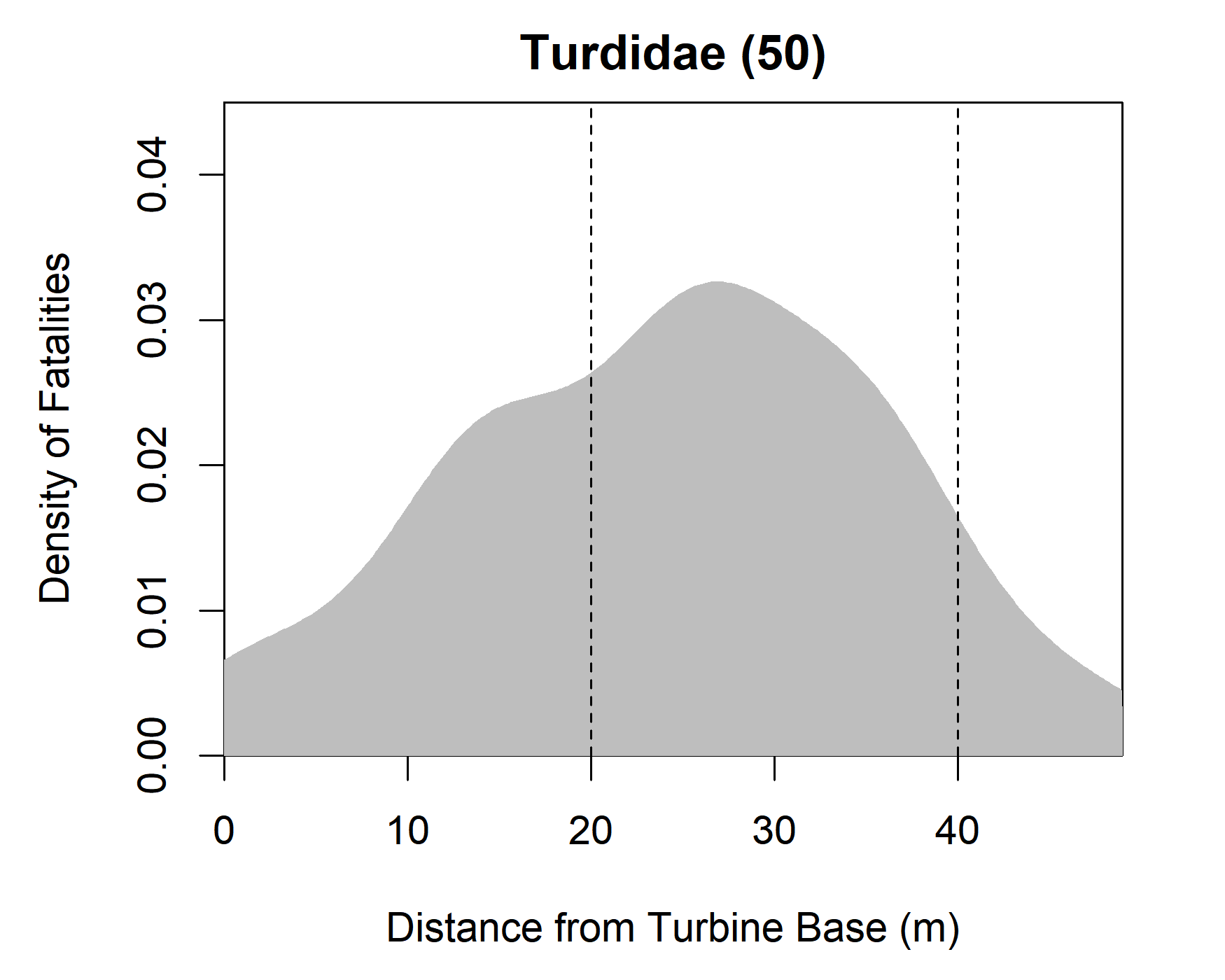

Supplement: S14 Fig — Data from publicly available reports and from reports submitted to the US Fish and Wildlife Service by 44 wind facilities in the Northeastern US. Sample size given in parentheses. (TIFF) [file pone.0238034.s015.tiff]

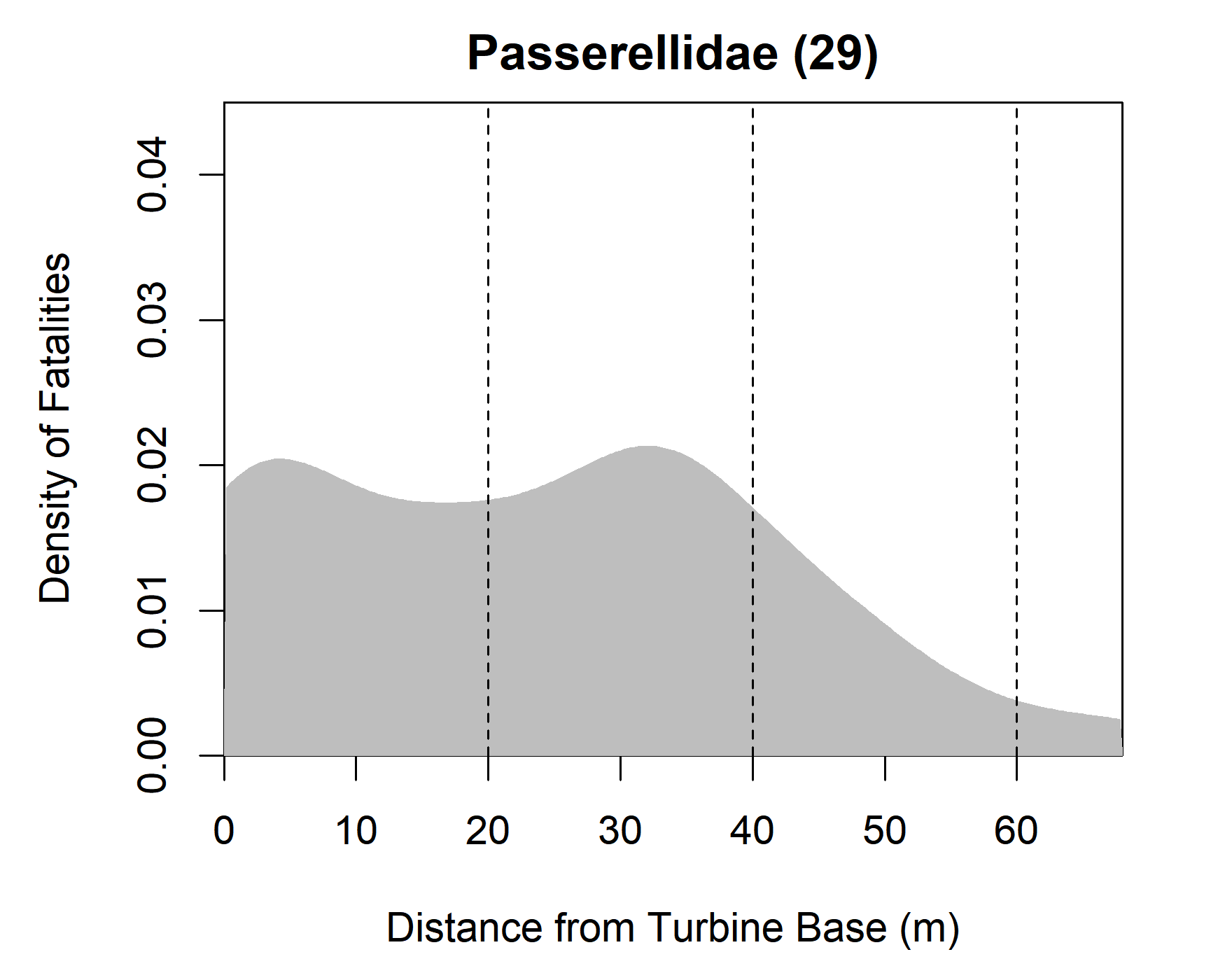

Supplement: S15 Fig — Data from publicly available reports and from reports submitted to the US Fish and Wildlife Service by 44 wind facilities in the Northeastern US. Sample size given in parentheses. (TIFF) [file pone.0238034.s016.tiff]

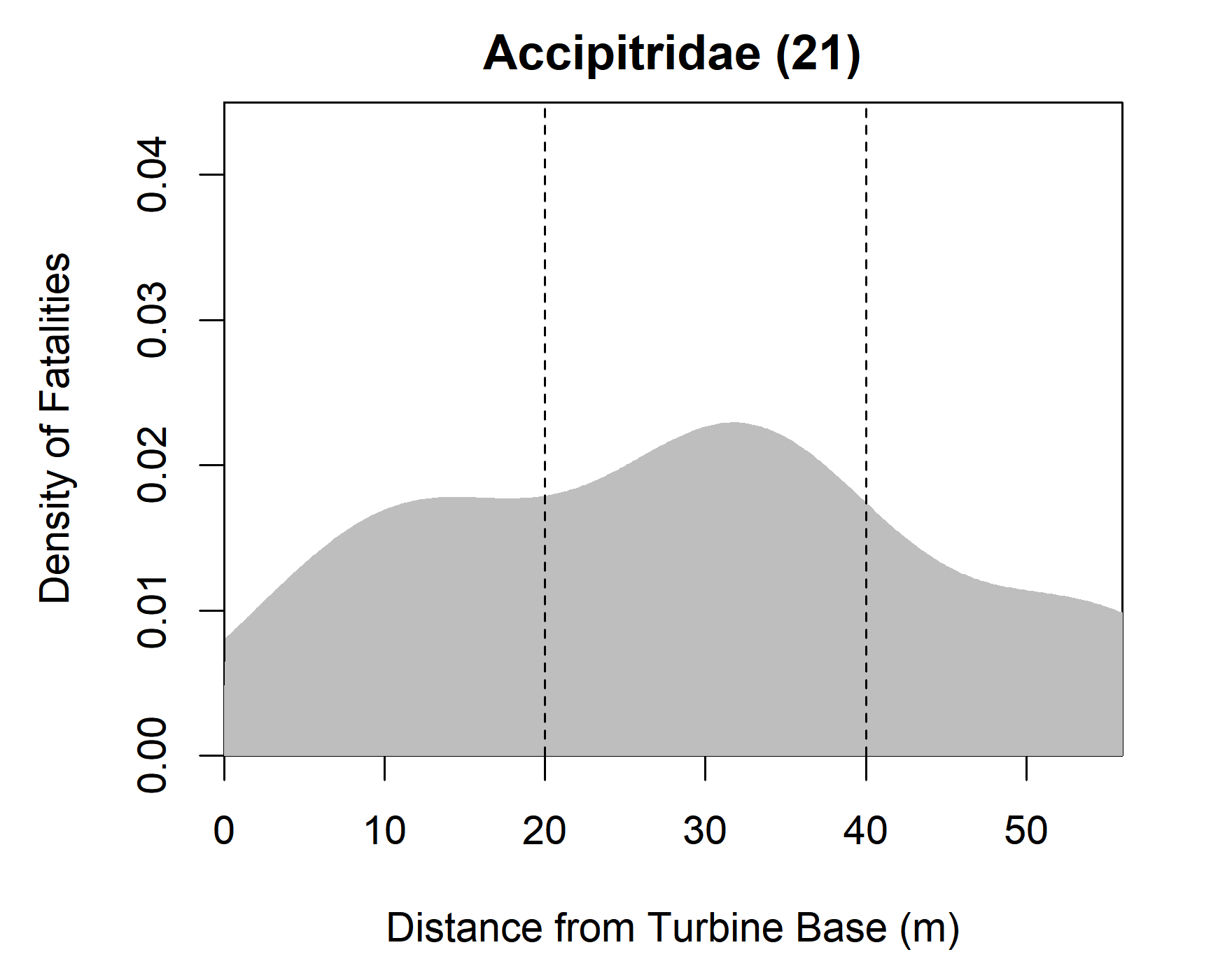

Supplement: S16 Fig — Data from publicly available reports and from reports submitted to the US Fish and Wildlife Service by 44 wind facilities in the Northeastern US. Sample size given in parentheses. (TIFF) [file pone.0238034.s017.tiff]

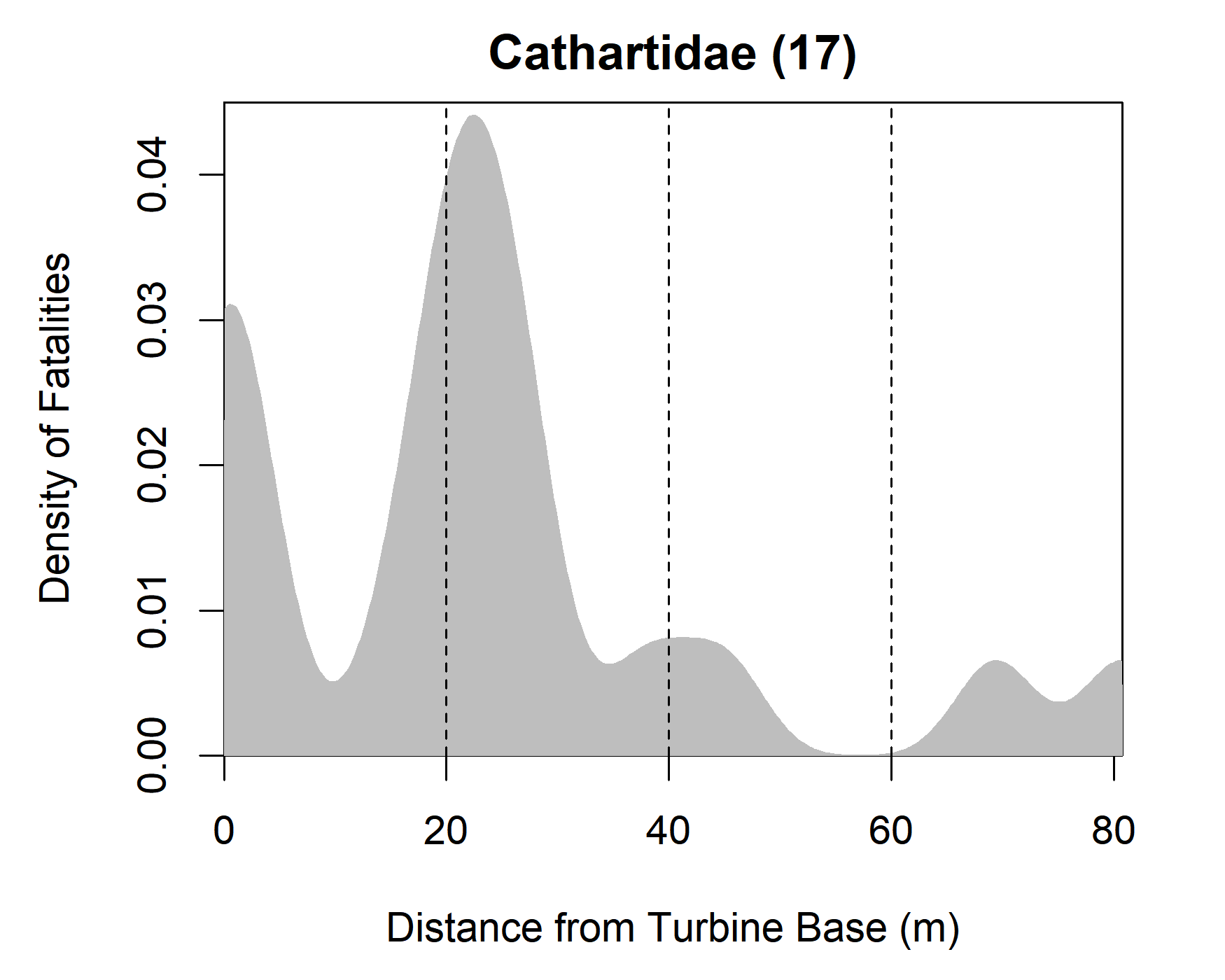

Supplement: S17 Fig — Data from publicly available reports and from reports submitted to the US Fish and Wildlife Service by 44 wind facilities in the Northeastern US. Sample size given in parentheses. (TIFF) [file pone.0238034.s018.tiff]

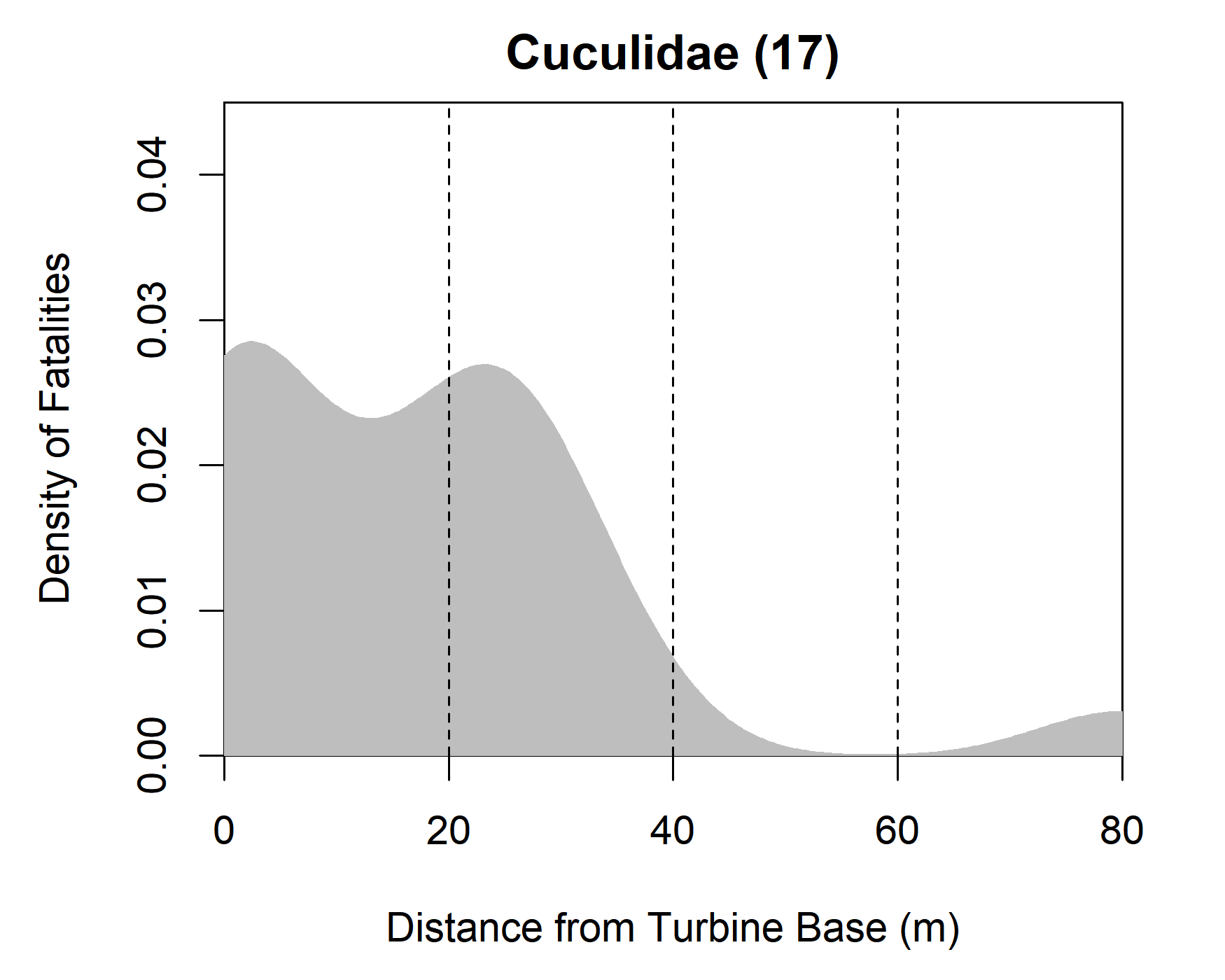

Supplement: S18 Fig — Data from publicly available reports and from reports submitted to the US Fish and Wildlife Service by 44 wind facilities in the Northeastern US. Sample size given in parentheses. (TIFF) [file pone.0238034.s019.tiff]

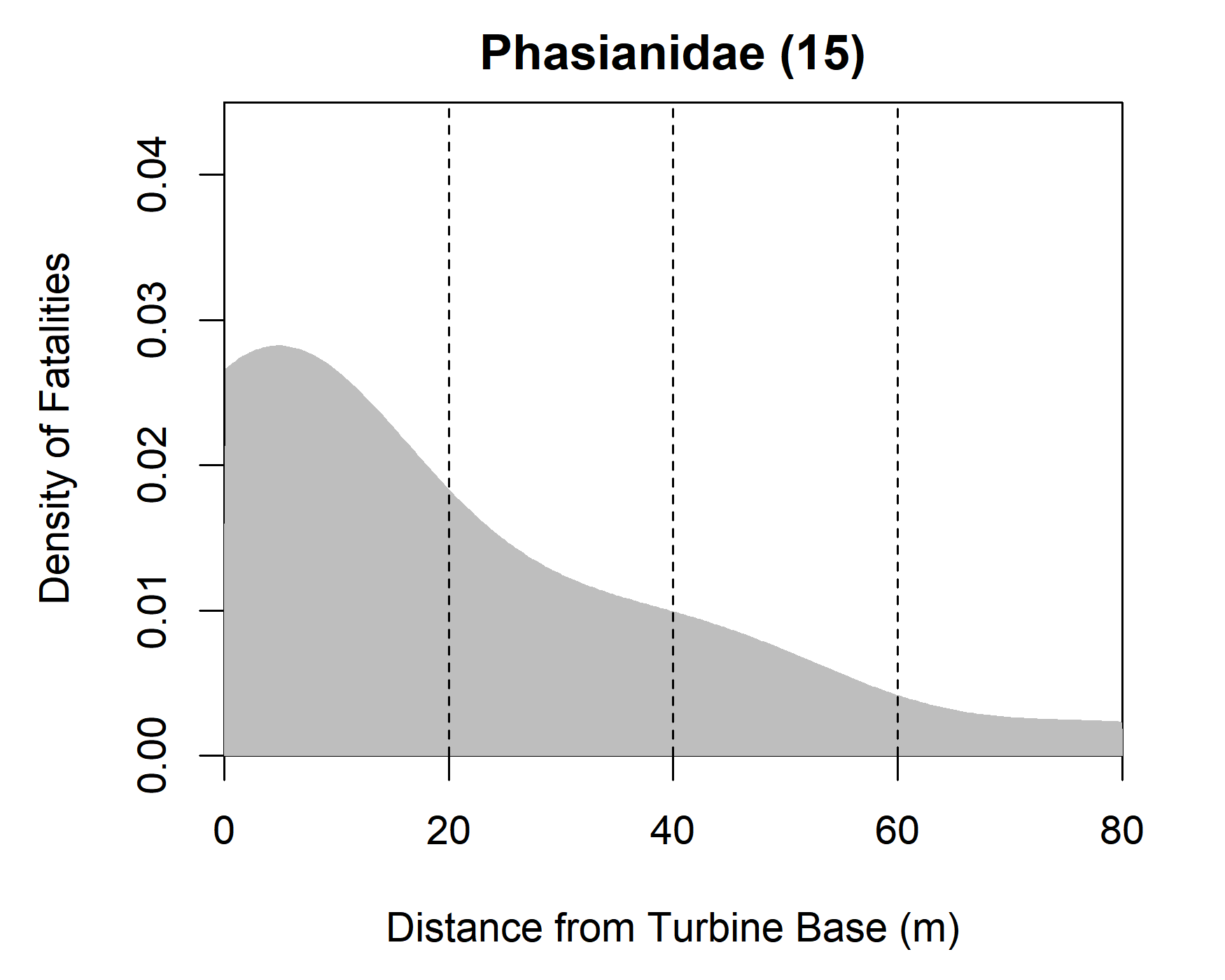

Supplement: S19 Fig — Data from publicly available reports and from reports submitted to the US Fish and Wildlife Service by 44 wind facilities in the Northeastern US. Sample size given in parentheses. (TIFF) [file pone.0238034.s020.tiff]

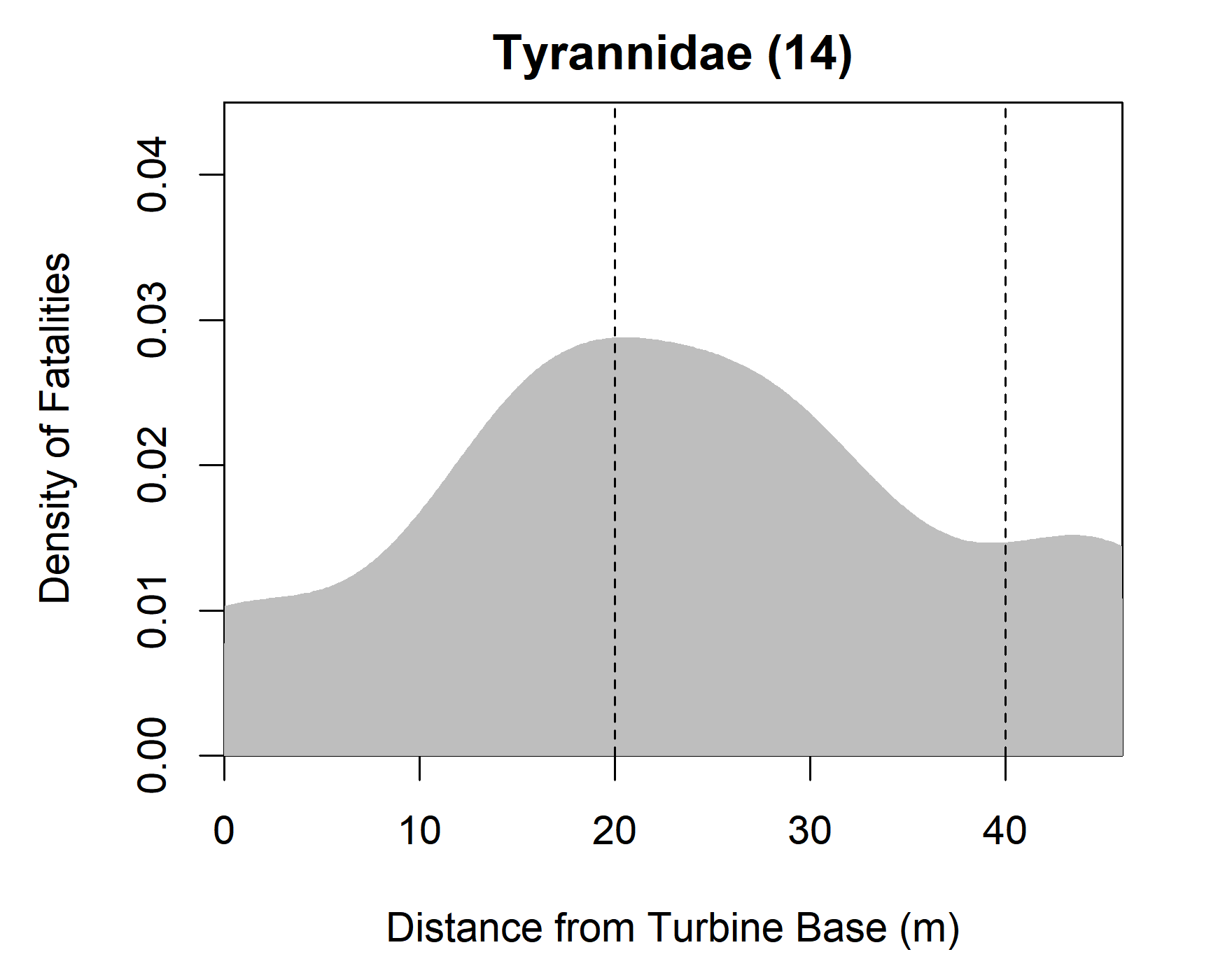

Supplement: S20 Fig — Data from publicly available reports and from reports submitted to the US Fish and Wildlife Service by 44 wind facilities in the Northeastern US. Sample size given in parentheses. (TIFF) [file pone.0238034.s021.tiff]
