# Supplementary material for: An evaluation of bird and bat mortality at wind turbines in the Northeastern United States
Source: PLoS One. 2020 Aug 28;15(8):e0238034. doi: 10.1371/journal.pone.0238034 (PMC7454995; doi:10.1371/journal.pone.0238034)
Supplement: S1 Table — (DOCX) [file pone.0238034.s024.docx]

**Table S24. Variables, number of parameters, delta Quasi-AIC (ΔQAIC), QAIC weights (*w_i_*), and log-likelihood (LL) for all-taxa full model set**. Shaded rows represent models excluded from averaging due to ΔQAIC > 6.

| **Variables^a^** | ***K*** | **ΔQAIC** | **LL** | ***w_i_*** |
| --- | --- | --- | --- | --- |
| A, M | 4 | 0.00 | -12838.36 | 0.28 |
| A | 3 | 0.74 | -12849.75 | 0.19 |
| A, D, M | 5 | 1.45 | -12836.08 | 0.13 |
| A, H, M | 5 | 1.99 | -12838.33 | 0.10 |
| A, D | 4 | 2.21 | -12847.56 | 0.09 |
| A, H | 4 | 2.73 | -12849.73 | 0.07 |
| A, D, H, M | 6 | 3.41 | -12835.89 | 0.05 |
| A, D, H | 5 | 4.16 | -12847.36 | 0.03 |
| A, D, H, M, D:H | 7 | 4.81 | -12833.42 | 0.03 |
| A, D, H, D:H | 6 | 5.57 | -12844.88 | 0.02 |
| M | 3 | 18.03 | -12921.67 | 0.00 |
| D, M | 4 | 19.55 | -12919.64 | 0.00 |
| H, M | 4 | 20.01 | -12921.56 | 0.00 |
|  | 2 | 20.17 | -12938.85 | 0.00 |
| D, H, M | 5 | 21.54 | -12919.61 | 0.00 |
| D | 3 | 21.71 | -12936.95 | 0.00 |
| H | 3 | 22.14 | -12938.76 | 0.00 |
| D, H, M, D:H | 6 | 22.91 | -12916.99 | 0.00 |
| D, H | 4 | 23.70 | -12936.90 | 0.00 |
| D, H, D:H | 5 | 25.07 | -12934.30 | 0.00 |

^a^Animal type (A), turbine rotor diameter (D), turbine hub height (H), mass (M), turbine diameter:height (D:H), migration distance (MD), migration timing (MT)
