# Supplementary material for: An evaluation of bird and bat mortality at wind turbines in the Northeastern United States
Source: PLoS One. 2020 Aug 28;15(8):e0238034. doi: 10.1371/journal.pone.0238034 (PMC7454995; doi:10.1371/journal.pone.0238034)
Supplement: S2 Table — (DOCX) [file pone.0238034.s025.docx]

**Table S25. Variables, number of parameters, delta Quasi-AIC (ΔQAIC), QAIC weights (*w_i_*), and log-likelihood (LL) for birds-taxa full model set.** Shaded rows represent models excluded from averaging due to ΔQAIC > 6.

| **Variables^a^** | ***K*** | **ΔQAIC** | **LL** | ***w_i_*** |
| --- | --- | --- | --- | --- |
| M, MD | 4 | 0.00 | -6126.48 | 0.18 |
| MD | 3 | 0.49 | -6137.83 | 0.14 |
| D, M, MD | 5 | 1.31 | -6123.32 | 0.10 |
| D, MD | 4 | 1.79 | -6134.63 | 0.07 |
| M, MD, MT | 5 | 1.95 | -6126.25 | 0.07 |
| H, M, MD | 5 | 1.96 | -6126.30 | 0.07 |
| MD, MT | 4 | 2.14 | -6136.22 | 0.06 |
| H, MD | 4 | 2.46 | -6137.68 | 0.05 |
| D, M, MD, MT | 6 | 3.26 | -6123.09 | 0.04 |
| D, H, M, MD | 6 | 3.30 | -6123.28 | 0.04 |
| D, MD, MT | 5 | 3.43 | -6133.00 | 0.03 |
| D, H, MD | 5 | 3.77 | -6134.55 | 0.03 |
| H, M, MD, MT | 6 | 3.91 | -6126.06 | 0.03 |
| H, MD, MT | 5 | 4.10 | -6136.05 | 0.02 |
| D, H, M, MD, D:H | 7 | 4.80 | -6121.03 | 0.02 |
| D, H, MD, D:H | 6 | 5.13 | -6131.66 | 0.01 |
| D, H, M, MD, MT | 7 | 5.25 | -6123.05 | 0.01 |
| D, H, MD, MT | 6 | 5.42 | -6132.94 | 0.01 |
| D, H, M, MD, MT, D:H | 8 | 6.76 | -6120.82 | 0.01 |
| D, H, MD, MT, D:H | 7 | 6.79 | -6130.11 | 0.01 |
| M | 3 | 13.03 | -6194.98 | 0.00 |
| D, M | 4 | 14.38 | -6192.05 | 0.00 |
| M, MT | 4 | 14.91 | -6194.43 | 0.00 |
| H, M | 4 | 15.01 | -6194.89 | 0.00 |
| D, M, MT | 5 | 16.26 | -6191.49 | 0.00 |
| D, H, M | 5 | 16.36 | -6191.93 | 0.00 |
| H, M, MT | 5 | 16.89 | -6194.34 | 0.00 |
| MT | 3 | 17.16 | -6213.83 | 0.00 |
| D, H, M, D:H | 6 | 17.99 | -6190.27 | 0.00 |
|  | 2 | 18.17 | -6227.53 | 0.00 |
| D, H, M, MT | 6 | 18.23 | -6191.37 | 0.00 |
| D, MT | 4 | 18.50 | -6210.80 | 0.00 |
| H, MT | 4 | 19.14 | -6213.75 | 0.00 |
| D | 3 | 19.53 | -6224.61 | 0.00 |
| D, H, M, MT, D:H | 7 | 19.86 | -6189.69 | 0.00 |
| H | 3 | 20.16 | -6227.49 | 0.00 |
| D, H, MT | 5 | 20.46 | -6210.65 | 0.00 |
| D, H | 4 | 21.47 | -6224.37 | 0.00 |
| D, H, MT, D:H | 6 | 21.96 | -6208.37 | 0.00 |
| D, H, D:H | 5 | 22.96 | -6222.03 | 0.00 |

^a^Animal type (A), turbine rotor diameter (D), turbine hub height (H), mass (M), turbine diameter:height (D:H), migration distance (MD), migration timing (MT)
