# Supplementary material for: An evaluation of bird and bat mortality at wind turbines in the Northeastern United States
Source: PLoS One. 2020 Aug 28;15(8):e0238034. doi: 10.1371/journal.pone.0238034 (PMC7454995; doi:10.1371/journal.pone.0238034)
Supplement: Attachment — Submitted filename: Response to reviewers.docx [file pone.0238034.s027.docx]

Dear Dr. Magar,

My coauthors and I are grateful for the feedback provided by the reviewers, which we feel has greatly strengthened our manuscript. Enclosed is our revised manuscript titled “An evaluation of bird and bat mortality at wind turbines in the Northeastern United States” (PONE-D-20-07129) that we wish to have considered for resubmission as an original research article in PLOS ONE. We believe that we have addressed the comments and concerns put forth by the reviewers. For example, we have revised the Introduction section, removing information not immediately relevant and adding two paragraphs which provide clear justification for our objectives. In both our Introduction and Discussion sections, we have made it more evident why results from our study are relevant to managers and researchers. Finally, we have adjusted our statistical methodology in accordance with reviewer suggestions. Below, we respond to each reviewer’s comments individually. We have kept the original line numbers associated with each comment but in our response have included line numbers that reference the updated manuscript.

This resubmission represents original work that is not under consideration for publication elsewhere. Data in this manuscript are not included in previous publications nor will they be submitted for publication elsewhere. We are willing to cover page chargers and any other costs associated with the publication of the manuscript. This paper aims to make a contribution to the fields of conservation science, ornithology, mammalogy, and animal behavior, by reporting on patterns associated with bird and bat mortality at wind facilities, with an emphasis on the spatial arrangement of carcasses. To our knowledge, our dataset represents the largest ever compiled for bird and bat mortality at wind facilities in the Northeastern United States, an area where wind energy production is rapidly increasing. In addition, our empirical support for bird collisions with turbines poles in addition to blades is novel and germane for scientists, managers, and those involved in the wind power industry.

Thank you for your consideration.

Sincerely,

Daniel Choi

No comments from the academic editor.

**Reviewer #1 General Comments**

General Comment 1

*Choi et al. present data on taxonomic composition and fall distances of bird and bat carcasses from wind-energy facilities in the northeastern United States. I have three overall comments, which are fleshed out further in my specific comments below. First, I found the manuscript generally hard to read. There was much extraneous text not directly related to the topic of this study.*

**Response:** Thank you for pointing out that we have included information not specifically relevant to this study. We have removed extraneous text from the introduction and discussion. For example, we removed four lines from the third paragraph (lines 59-74) of our introduction, which focused on bats. Additionally, we removed paragraphs in our introduction and discussion which mentioned mitigation efforts. We also completely rewrote our implications section so that it clearly reflects results from the present study.

General Comment 2

*Second, I found objective 1 (species composition) to be the weakest part of this manuscript because this is a topic that has already been well studied and the results the authors show are already well known (which is consistent with the authors repeatedly saying that their “results confirm prior studies”). It is sometimes appropriate to repeat and validate prior studies, but the authors didn’t provide a justification as to why they thought this was necessary to do. Furthermore, it isn’t clear to me how much of the data they present on the species composition of carcasses has already been published in prior studies.*

**Response:** Thank you for pointing this weakness out. We have revised our introduction to provide justification for examining species composition (objective 1) and have clarified how our data differs from what is currently published. Prior to this study, the two most significant reviews of mortality, and thereby species composition, were those by Erickson et al. in 2016 [17] and the American Wind Wildlife Institute in 2019 [34]. These studies summarized mortality by region. For example, their eastern North America region extended from eastern Texas to New York. Within this region, Erickson et al. and AWWI included data from eight and 20 wind facilities. This is a relatively small number considering the geographic extent of this region. Additionally, each of these studies specifically called for improved reviews to corroborate their findings. For example, AWWI specifically referred to their results as “tentative.” Therefore, we are able to offer substantially improved picture of species composition in the Northeastern US where we look at data from 44 wind facilities. Due to confidentiality of reports, we cannot be sure how many reports in our dataset overlap with that of AWWI. However, because we are able to include so many more facilities than AWWI, we are confident that at least most of our data is from facilities not included in their report.

General Comment 3

*Finally, in regards to objectives 2-3 (on fall distances): these results may be greater interest to readers since there hasn’t been as much study of this topic. However, although this topic has been less studied, the authors didn’t convince me why it is important to understand. I also found their discussion of how wildlife managers could use such results to be vague and hand wavy.*

**Response**: Thank you for making us aware of these points regarding support for objectives 2 and 3 and our discussion. We revised our introduction and discussion and believe it is now much stronger. First, we have combined objectives 2 and 3 into a single objective which is now to “examine whether animal type (bird/bat), body size, migration behavior, or turbine size influence the fall distances of carcasses associated with turbines” (lines 109-110). We have added a paragraph (lines 75-93) to our introduction that explains why understanding the patterns and sources of variation in fall distance are important to designing effective search protocols and accurately estimating mortality. We have also revised our discussion to reflect this reasoning. For example, we point out that for species such as vireos, which have sharp peaks in their distance distribution near the turbine base, mortality may potentially by overestimated and that estimates must account for skewed distance distributions (lines 449-452).

**Reviewer #1 Specific Comments**

*Lines 17-19: It would be helpful if the authors explained why an understanding of the spatial patterns of fatalities would be useful. What problem would such data help to solve? How would wildlife managers, etc. use such data?*

**Response:** We have added a sentence to our abstract explaining why this is important: “Understanding how far carcasses typically fall from turbines is important in designing effective search protocols and in estimating total mortality” (lines 19-20). Additionally, we have added a paragraph (lines 75-93) to our introduction that explains this in detail.

*Lines 30-31: I’m not sure it’s fair to say that it is thought that turbines are a threat only because of their motion. Certainly, it is well known that other stationary structures can cause bird fatalities.*

**Response:** Thank you for pointing this out. Certainly, this is well known and it is intuitive that turbine poles are a source of mortality. Yet, to our knowledge, this has not been discussed in the literature. There are a few sparse mentions in reports of gamebirds which may collide with poles. However, this has not been discussed for other bird groups. Many papers and reports explicitly refer to only turbine blades. We have adjusted our abstract and discussion to better communicate this point. For example, our discussion paragraph on lines 453-465 begins with “The concept of turbine pole collisions is not novel, as bird collisions with other tall structures (e.g., communication towers, buildings) are very common [2]. However, apart from brief discussion of collisions by upland gamebirds [6], pole collisions across avian taxa have received very minor attention by either researchers or management agencies” (lines 453-456).

*Lines 31-32: Is this a finding of the present manuscript or prior studies? It sounds like the authors are referring to prior studies since they don’t present data in the abstract related to this already well-documented topic. However, it doesn’t make sense to have the conclusion of the abstract related to prior studies. The conclusion should be related to the present study.*

*Lines 33-34: This sentence is vague and much too general. This abstract doesn’t seem to interpret the results that this study presents. Instead of this sentence, I suggest the authors provide an interpretation or discuss the implications of the results of their results regarding fall distance, body mass, turbine height, etc.*

**Response:** Thank you for pointing out that the last two sentences (both lines 31-32 and 33-34) of our abstract do not reflect the conclusions of the present study and thank you for providing suggestions. We have altered the end of our abstract so that it ends with: “Bird and bat fall distance distributions were notably multimodal, but only birds exhibited a high peak near turbine bases, a novel finding we attribute to collisions with turbine poles in addition to blades. This phenomenon varied across bird species, with potential implications for the accuracy of mortality estimates. Although pole collisions for birds is intuitive, this phenomenon has not been formally recognized. This finding may warrant an updated view of turbines as a collision threat to birds because they are a tall structure, and not strictly as a function of their motion” (lines 29-35). Additionally, we included in earlier sentence regarding turbine size and fall distance: “This suggests that as turbines increase in size, a greater search radius will be necessary to detect carcasses” (lines 28-29).

*Line 40: A good recent citation to add here and elsewhere in this manuscript is Allison et al. 2019. Impacts to wildlife of wind energy siting and operation in the United States. Issues in Ecology. Report number 21.*

**Response:** Thank you for this valuable source. We have included this paper as a reference here and elsewhere throughout the manuscript.

*Line 59: Is this citation correct? Perhaps add additional citations here to support the sentence on lines 57-59.*

**Response:** Thank you for catching this incorrect citation. We have modified this sentence to refer to raptors only and we have added additional references: “There is particular concern regarding raptors of low abundance [2, 18, 19], whose low reproductive rates and high adult survival may hamper recovery from mortality at wind facilities [6, 20]” (lines 53-55).

*Lines 59-62: This is probably true for most bird species, but might not be true for some such as raptors (Allison et al. 2019; Katzner et al. 2020).*

**Response**: Thank you for this comment. Certainly, some species may be severely impacted by collision with wind turbines. We have modified the beginning of this sentence: “However, for most bird species, wind turbines contribute a small amount to total anthropogenic mortality [21] and may not significantly impact vital rates (e.g., annual survival probability) and state variables (e.g., abundance, density, occupancy) at the population level” (lines 55-58).

*Line 63: This sentence is too general. Wind-energy fatalities of some bird species are of concern, and some species of bats aren’t thought to be impacted by wind turbines.*

**Response:** We have modified this sentence to specifically refer to differences between passerine birds and migratory tree bats; “Compared to many passerine birds, wind turbines may have a more pronounced negative impact on migratory tree bats” (lines 61-62).

*Lines 64-67: I’m not sure I agree with the logic of these sentences. Bats are more active at low than high wind speeds, but has that been shown to be the cause of slightly higher bat than bird mortality in the eastern US as these sentences imply? Is there a reference the authors could cite to support this claim?*

**Response:** Thank you for pointing out the error in this argument. We have removed the second sentence which links increased bat mortality to lower wind speeds in the east.

*Lines 69-71: True, but the authors should acknowledge (since some readers won’t know) that the tree-roosting bats (i.e., eastern red, hoary, and silver-haired bats) that experience the greatest numbers of fatalities at wind-energy facilities don’t seem to be affected by WNS.*

**Response:** Thank you for this suggestion. We have added the following sentence: “However, it should be noted that migratory tree bats that experience the most mortality at wind facilities are not as affected by white-nose syndrome [26]” (lines 70-71).

*Lines 71-72: I’m not sure what this sentence means. Certainly bats are killed at wind-energy facilities in regions of the U.S. where WNS occurs. Please clarify.*

**Response:** In an effort to reduce the introduction following recommendation by Reviewer 1, this sentence has been deleted.

*Lines 76-79: True, but this sentence is tagged on to the end of this paragraph and its relevance to this paragraph and study is unclear.*

**Response:** This sentence has been removed.

*Lines 86-87: This is the topic of this paper, but it wasn’t mentioned until the 5th paragraph of the Introduction. Most readers won’t have the patience to wade through 5th paragraphs of background material that is only indirectly related to the topic of this manuscript.*

**Response:** Thank you for comment regarding the length and detail of the introduction. We attempted to write our manuscript in a standard general-to-specific format. We have reduced the first three paragraphs and removed the fourth. This is now paragraph four. Several of our reductions were the result of incorporating Reviewer 2’s specific comments for this Section. It may be important to note that not only did Reviewer 3 not take issue with the writing style/flow of the manuscript, they stated “This is an excellently written manuscript on factors associated with wind turbines that cause mortality in both birds and bats. I really enjoyed reading this manuscript and the editing is one of the best that I have encountered over my many years as a peer reviewer. So, kudos to the authors on such excellent prose.”

*Lines 92-95: This (i.e. “dynamics and nuances”) is much too vague. The authors should be clear and specific about why/how information on the spatial arrangement of carcasses would benefit monitoring, mitigation, and conservation.*

**Response:** We have greatly expounded this paragraph (lines 75-93), removed vague language, and included clear and specific sentences regarding the importance of studying spatial arrangement of carcasses. Specifically, we have focused our discussion on fall distance and have explained how this is importance in designing carcass search protocols and in accurately estimating mortality.

*Lines 97-98: Objective 1 is something that we arguably know a lot about already (e.g. Allison et al. 2019, AWWI reports that are cited, many other peer-reviewed publications and consultant reports). If the authors truly feel that this is a topic that hasn't been adequately address then their Introduction should articulate that what their study will address/accomplish that hasn’t been addressed/accomplished in prior studies.*

**Response:** Please see our response to an identical comment, Reviewer 1 General Comment 2.

*Lines 98-99: How is fall distance defined and why is it important if it varies between bats and birds? How might the authors expect it to vary? As with their objective 1, the Introduction hasn’t set up this objective.*

**Response:** Concerning objective 2, please see our response to an identical comment, Reviewer 1 General Comment 3. We have adjusted our introduction to answer these questions. Regarding the definition of fall distance, we have included a definition in the second sentence of the fourth paragraph of the introduction: “the horizontal distance between a carcass and the nearest turbine pole” (lines 76-77).

*Lines 99-100: Why do the authors expect these factors might influence spatial arrangement of carcasses?*

**Response:** Please see our response to an identical comment, Reviewer 1 General Comment 3. We have adjusted our introduction to answer these questions.

*Line 110: What is meant by “migration status”?*

**Response:** We have modified this sentence with parentheses following: “By species, we added migration status (i.e., resident, migratory, partial migrant)…” (line 128).

*Lines 130-131: What is the justification for censoring/removing records from non-migratory species?*

**Response:** Removing non-migratory birds was necessary to conduct likelihood ratio tests between models during our original stepwise model fitting. Had we left non-migratory birds in our analysis, those models with migratory-related variables would have had fewer data points (non-migratory species’ values being ‘NA’) than those without these variables, invalidating statistical comparison between models. Although we have changed our model fitting to an Information-Theoretic approach based off the recommendation of Review 3, missing data similarly confound model comparison in this approach. In revising our methodology, we have explained the model comparison justification for removing non-migratory birds (lines 136-151) and included a relevant citation (Nakagawa and Freckleton 2010 [47]).

*Lines 202-203: I know what the authors mean, but fog isn’t a direct cause of fatalities.*

**Response:** Thank you for pointing this out. We have revised this to indicate that fog was associated with mortality events, but did not specifically cause mortality: “Reports indicated that the largest event (80 fatalities) was associated with heavy fog” (lines 235-236).

*Lines 232-237: What do the error bars in panel A indicate? What do the small tick marks in panel B and the thicker black bars in panel C indicate? Also, what do lower vs. higher cHeight values indicate (lower values = shorter towers?)?*

**Response:** We have removed this figure. Following on the recommendation of Reviewer 3, we revised our methodology and fit our models according to an Information-Theoretic approach. The results showed several viable candidate models for the all-taxa, birds-only, and bats-only sets, leading us to perform model-averaging. Unfortunately, we were unable to find current software implementation for plotting model-averaged coefficients in a manner similar to our original response curves. However, we have added a table with model-averaged coefficients, standard errors, and 95% confidence interval, which we believe comparably conveys the direction and magnitude of the variable responses.

*Lines 265-267: Although statistically significant, I wonder if the difference in the two modes of bat fall distance are biologically significant.*

**Response:** Thank you pointing this out. We agree, and have included the following sentence in the first paragraph of our discussion section titled “Differences between Birds and Bats”: “Bats displayed two peaks, either side of 20 m, that were comparable in density and likely not biologically significant” (lines 417-418).

*Line 273: Is the fact that the results of this study vary little from existing literature because many of the same data were used in this study as in prior studies? It would be helpful if somewhere in the manuscript (probably the methods) the authors addressed if/how the datasets used in the present manuscript differ from previously published datasets.*

**Response:** Please see our response to an identical comment, Reviewer 1 General Comment 3. We have adjusted our introduction to answer these questions. Additionally, we have added the following to the first paragraph of our methods section: “We are unsure how many of these facilities may have also contributed data to studies such as those by AWWI [22, 34]. However, we include 44 total wind facilities, compared to 20 in the eastern region of North America included by AWWI [34]. Thus, the majority of wind facilities in our study contribute new data in examining species composition” (lines 117-121).

*Lines 317-320: I’m not sure that this is true. What evidence supports the statement that use of generalized body mass values creates greater uncertainty than categorical classifications. I imagine there can be a great deal of variation in a factor such as body mass.*

**Response:** Thank you for your comment. Undeniably, the mass of one individual is not the mass of all members of that species. This metric will vary within species based on sex, age, condition, etc. However, previous studies have used subjective categorizations of large and small, either based on mass and/or length [27, 34]. AWWI 2019 [34] divided large and small birds as having a total length of greater or less than 30 cm. We think our method of using average species masses improves greatly over just using two very broad categories. We have added a sentence mentioning this to the fourth paragraph of our introduction (lines 86-89). We have additionally modified the first paragraph of our discussion section titled “Spatial Patterns” to address this point (lines 361-377).

*Lines 320-322: This sentence implies that post-construction mortality studies often focus on finding carcasses of a particular species, but I’m not sure that’s the case. They are typically interested in finding all bird or bat (or both) carcasses, as I understand.*

**Response:** We have modified this sentence to suggest that particularly for large-bodied birds, search protocols must ensure they have a wide enough search radius: “A practical implication is that post-construction mortality studies concerned with large-bodied birds (e.g., raptors) should consider implementing wider search radii [27] or adjusting their weighting of area searched during analysis to reflect the increased potential of discovering larger species farther from turbines” (lines 370-374). Further, we have modified this paragraph to reiterate that our finding that mass is positively associated with fall distance corroborates findings from prior models.

*Lines 322-325: Figure 5 in the 2019 AWWI report shows similar fall distances for small and large birds. Why might the results of the present study differ from that report?*

**Response:** Thank you for drawing our attention to this figure. We have added a sentence to this paragraph (first paragraph of Discussion: Spatial Patterns) discussing this question: “Moreover, while AWWI’s [34] distance distributions for large and small birds did not appear to differ, this may have been the result of overly broad size bins” (lines 369-370).

*Lines 331-333: Please specify how this finding would be relevant to post-construction studies.*

**Response:** We have removed this sentence and have suggested that both “The positive association we found between bird fall distance and turbine rotor diameter and the additional finding that fall distance for birds and the all-taxa group increased with the interaction of increasing rotor diameter and hub height validate prior models and experiments suggesting that turbine height and blade length [27, 33] are positively related to fall distance” (lines 378-381).

*Lines 339-340: Correct. Facility ID is very important, which implies that it will be difficult to make management recommendations (as the authors do earlier in the text) without an understanding of what it is about different facilities (or survey techniques, etc., among facilities) that influences fall distance.*

**Response:** We have modified the ending of this paragraph (third paragraph of Discussion: Spatial Patterns) with: “Despite this variation, managers can still expect findings such as the positive relationship between turbine size and fall distance to be generally true at specific facilities” (lines 401-402). We agree that facility to facility variation prohibits very specific management recommendations. However, our general findings (e.g., fall distance positively influenced by mass, turbine size, etc.) can still be helpful in providing *general* predictive power, regardless of the variation at specific facilities.

*Lines 368-392: These are interesting findings.*

**Response:** Thank you for your interest.

*Lines 393-402: We know that mortality at wind-energy facilities is relatively minor relative to other sources of mortality (cats, buildings) for most species of birds, so I’m not sure that managers who want to reduce bird fatalities would really pay much attention to whether fatalities at wind-energy facilities are caused by the pole or blades.*

**Response:** We agree that managers may not pay specific attention to whether individual fatalities are caused by collisions with poles or blades. Because of this very fact, in combination with evidence for frequent pole collisions, we suggest that simply that managers may not need to focus specifically on the threat of moving turbine blades for many species. Instead, they should begin to view turbines as more of a threat simply because they are large structures like other towers. We have modified this paragraph (lines 453-465) accordingly. Even so, we think that managers’ interest in mitigating any collisions is evident in the large body of guidance developed by state and federal agencies, and in the intense efforts of wind projects themselves to understand this issue. Any understanding that could help carve away potential collisions is of interest to wind energy companies.

*Lines 393-395: Do these studies really make this assumption? It is well known that birds are killed by other tall structures, so I find it odd that these studies would assume that bird mortality at wind-energy facilities is primarily attributable to collision with blades.*

**Response:** Please see our response to an identical comment, Reviewer 1 Specific Comment Lines 30-31.

*Lines 403-414: This paragraph doesn’t seem very related to the results presented in this manuscript.*

**Response:** Thank you for pointing this out. This paragraph has been removed.

*Lines 427-428: How many? It feels like this is a point the authors should have made in their Introduction.*

**Response:** We have added comments on this matter to the introduction (lines 94-106) and the methods (lines 112-121). We also discuss this in our response to Reviewer 1 General Comment 2.

*Lines 437-451: None of these implications are derived from the results presented in this manuscript.*

**Response:** Thank you for pointing this out. We have completed rewritten our implications section (lines 487-497) ensuring that they are clearly derived from the results in this manuscript.

**Reviewer #2 General Comments**

General Comment 1

The authors present a paper that looks at bat and bird fatalities at wind turbine facilities and runs a series of GLMMs to explore whether turbine specifications and species attributes such as mass or migratory status impact the distance at which the carcasses are found from the base of the turbine. Overall I believe this is worthy of publication and is a useful exploration that could help managers make regulatory decisions on how to mitigate bird and bat deaths at wind farms. I have several comments on the writing and some of how the analysis is presented, detailed below.

**Response:** Thank you for your support of this paper. We appreciate your comments regarding the writing and analysis and respond to them individually below.

**Reviewer #2 Specific Comments**

*Line 36: This paragraph is about how wind facilities impact wildlife, so it seems awkward to begin by mentioning how much wind power is growing. Instead, it would make more sense to begin the paragraph with something like "Wind turbine facilities can have direct and indirect negative impacts on wildlife.... then, after you've summarized the literature on different species responses to wildlife, you can end the paragraph with the fact that turbines are increasing across the world, tripling in the last decade, and therefore are expected to have further impacts on wildlife. I think the second and third paragraph should either be combined, or the two paragraphs should be separated by birds and bats, respectively. Jumping back and forth especially in the second paragraph is confusing for me as a reader.*

**Response:** Thank you for your advice. We have followed your suggestion and moved the first sentence to the end of the paragraph (lines 46-47). We have additionally separated the second (lines 48-58) and third (59-74) paragraphs by birds and bats.

*Line 66: I think rotor-swept zone should be defined here, it is not common knowledge what this is. Additionally, please define what speed "lower wind speed" is - what is the average wind speed considered low?*

**Response:** Because we do not have sources for the argument presented here, we have removed this sentence, including both references to “rotor-swept zone” and “lower wind speeds.”

*Line 69: white nose syndrome has been around for around 15 years now, I am not sure calling it 'recent' is accurate. Perhaps you could say something like "Since 2006, the fatal fungal pathogen white-nose syndrome has caused massive population declines in once-common bat species in the northeastern and midwestern US. Additional mortalities from wind turbine collisons could impact recovery of these species.."*
**Response:** Thank you for your suggestion. We have modified this sentence to: “Further, since 2006, many bat species have been severely impacted by white-nose syndrome and additional mortality from wind facilities could impact recovery of species of special concern [25]” (lines 68-70).

*Line 71: Please explain why that is. I am assuming it is because WNS deaths are so high, it is difficult to tease wind turbine deaths apart from that, right?*

**Response:** In an effort to reduce the introduction as suggested by Reviewer 1 we have removed this sentence.

*Line 72: an additional note: hoary bats are not particularly impacted by WNS as it mostly kills cave hibernating bats, of which hoary bats are not. But a reader not savvy on bats may see the sentence beforehand and this one and say, wait a minute - didn't you just say we couldn't tease apart wind turbine deaths from WNS deaths? This discrepancy should be cleared up in the paragraph.*

**Response:** We have added the following sentence to address this: “However, it should be noted that migratory tree bats which experience the most mortality at wind facilities are not as affected by white-nose syndrome [26]” (lines 70-71).

*Lines 80-85: This is a 2-sentence paragraph and I am unsure what the exact purpose of the paragraph is. I think if it remains in the paper it needs to be fleshed out a little more, maybe describe additional mitigation efforts that are not as effective?*

**Response:** This paragraph has been removed.

*Line 95: I think it would be helpful here to describe exactly why investigating the spatial arrangement of fatalities will benefit mitigation/conservation. "dynamics and nuances" is a little vague. Can you mention some specific things it could address?*

**Response:** We have greatly clarified this statement. This was also mentioned by another reviewer and we have added an entire paragraph (lines 75-93) to address this.

*Line 96: not super important but ~2000 data points is not considered large by some.. consider deleting – you could also call it long-term as it is from 2008-2017*

**Response:** We have removed the adjective “large,” and the sentence now reads: “Here, we investigated a dataset of bird and bat fatalities at wind facilities located in the Northeastern US” (lines 107-108).

*Line 100: spatial arrangement is still a sort of vague term to me.. is it fall distance? if so, perhaps that can go in parentheses after 'spatial arrangement'*

**Response:** We have removed the term “spatial arrangement” and simply referred to “fall distances” instead (line 110).

*Lines 102-109: This entire paragraph is written in passive tense which I find to be a bit awkward. Additionally, instead of listing out each state I think it would be much better to have a map highlighting the states where data came from and if possible, areas where wind turbines are located within the state. I recognize the latter may not be possible due to privacy concerns and endangered species location reporting, but a map at least showing the states would be beneficial especially for those readers not familiar with the USA.*

**Response:** We have changed the tense of the paragraph (lines 112-121) to active voice, except for the first sentence (lines 112-113). But because the authors had little or no role in receiving fatality data, we stated that “the USFWS received records” in order to explicitly communicate that these records were submitted to the agency as a whole, and not to individuals. Additionally, thank you for your recommendation to include a map. We have included a map (Fig. 1; lines 122-126) showing the Northeast within the United States, the amount of fatalities reported from each state, and the locations of wind facilities present in the Northeast. We were not able to include locations of wind facilities in this study due to privacy concerns.

*Line 110: This paragraph needs a topic sentence. Perhaps something like, "We supplemented the fatality data records with information that would serve as predictor variables or ways to subset data for our models"*

**Response:** We have added a topic sentence and altered the second sentence. The first two sentences now read: “We supplemented fatality records with classification information and model covariates. By species, we added migration status (i.e., resident, migrant, partial migrant) and population data [35], taxonomic classification information [36, 37], trophic guild information [38], timing (nocturnal/diurnal) and distance (long/short) of migration [39], and body mass [37-39]” (lines 127-130).

*Line 122: I'm confused why a Poisson distribution was used with fall distance rounded when fall distance could have been used in a Gaussian GLMM just as easily? Is it because you wanted to avoid predicting negative values at all? If so this should be explained here.*

**Response:** As shown in the density figures for birds and bats, fall distance approximates a Poisson distribution. Using a Gaussian link would have required the added and unnecessary step of transforming the fall distance data and may have led to poorer model performance (O’Hara & Kotze 2010).

O’hara RB, Kotze DJ. Do not log‐transform count data. Methods in ecology and Evolution. 2010 Jun 1;1(2):118-22.

*Line 157: I guess I am confused as to what was done with the estimated location of these modes? I understand that you identified them, but I'd like some detail as to why identifying multi-modality is important for the analysis, what it could do to inference, and what was done to address it.*

**Response:** The identified modes allowed us to stronger inference that rotor blades and towers are two distinct sources of mortality at wind facilities for birds. Because of the novel nature of this finding, we felt it important not rely on visual observation of the distributions alone. It is also our hope that by offering this methodology, we may encourage future comparison of our results against other datasets and study areas

*Line 192: Vespertillionidae are the only bats that exist in the regions you have data from*

**Response:** We have removed reference to *Vespertillionidae* and the sentence now reads: “Five species were identified and three species of migratory tree bats, hoary, silver-haired (*Lasionnycteris noctivagans*), and eastern red (*Lasiurus borealis*), accounted for over 90% of bat fatalities (Table 4)” (lines 226-228).

*Line 211: Just a preference, but censored is a little awkward. Consider changing it to "removed"*

**Response:** Another reviewer also mentioned this and throughout the paper, “censored” has been replaced with “removed.”

*Figures 3&4: I'd really like to have the response curves be shown on back-transformed variables so that it is easier to interpret these figures... right now I have no idea what the average bird or bat mass was, so these figures don't say much to me. If I saw the back-transformed masses, I could easily see the relationship and interpret it without trying to think about what the average mass is.*

**Response:** We have removed these figures. Please see our response to a similar comment regarding figure interpretation, Reviewer 1 Specific Comment Lines 232-237.

**Reviewer #3 General Comments**
This is an excellently written manuscript on factors associated with wind turbines that cause mortality in both birds and bats. I really enjoyed reading this manuscript and the editing is one of the best that I have encountered over my many years as a peer reviewer. So, kudos to the authors on such excellent prose. The only big issue I had with the manuscript was the flawed statistical approach. Other than that, this manuscript will make an excellent contribution to the literature once the statistical approach is revised. Here are three general comments on the methods followed by my line edits.

**Response:** We are very grateful for your praise for this paper. We have addressed your comments regarding our statistical approach in our responses below.

General Comment 1
*How is fall distance defined? Is this the perpendicular distance from the center of the hub to the ground or does this include both the vertical and horizontal distances from the hub? The authors skim over this like it is common knowledge. Likewise, they include the term “nacelle” in Table 1 but do not mention that term, nor define it in the Methods.*

**Response:** We have added the following definition of fall distance to the fourth paragraph of our introduction: “the horizontal distance between a carcass and the nearest turbine pole” (line 77). We have removed mention of the term “nacelle.”

General Comment 2
*The authors used wind facility as a random effect but how does this account for the number of turbines at the facility. Is turbine not nested within site? In particular, by including turbine ID as a random effect, the authors would not have to worry about the bias associated with assigning the facility with the most common hub height and rotor diameter.*

**Response:** We readily agree that nesting turbine ID as a random effect within facility ID would likely improve model performance. However, it is an unfortunate reality of data reporting that many carcass records with fall distance information do not also include associated turbine ID. It is primarily for this reason that we have not attempted such nesting of random effects. Regarding the influence of the number of turbines at facilities on fall distance, we believe that the random effect for facility ID may still address this variation. The total number of facility turbines is a factor that, like general landscape and habitat, may vary between facilities, but not within facilities. Therefore, if the number turbines at facilities does have relevance to fall distance, it is captured in the facility ID random effect.

General Comment 3
*The stepwise method of reducing model size has been discounted by many authors. Here are two examples of papers that show why this approach is longer acceptable. An information-theoretic approach using AIC would be much more appropriate in this context. As a result, I am unable to assess the Results section because of this flawed approach, unfortunately.
Mundry, R. & Nunn, C.L. (2009) Stepwise model fitting and statistical inference: turning noise into signal pollution. Am Nat, 173, 119-123.
Whittingham, M.J., Stephens, P.A., Bradbury, R.B. & Freckleton, R.P. (2006) Why do we still use stepwise modelling in ecology and behaviour? Journal of Animal Ecology, 75, 1182-1189.*

**Response:** Thank you for offering this critique. After consulting the referenced literature for background, we have fit our models according to an Information-Theoretic approach, and provided citations for our decisions on selection information criterion, delta threshold, and model averaging (lines 157-174).

**Reviewer #3 Specific Comments**

*Line 15: The authors should be more specific about who these benefits apply to. Presumably to humans but not to wildlife.*

**Response:** We have updated the sentence to specifically mention humans: “Wind energy offers substantial benefits to humans, but wind facilities can negatively affect wildlife, including birds and bats” (lines 15-16).

*Line 24: Unnecessary to include “Generalized linear mixed models revealed that”. Simply begin with “Turbine size…”*

**Response:** We have removed “Generalized linear mixed models revealed that” from the sentence (lines 27-28).

*Line 53: Remove “For bats,” since it is redundant with the rest of the sentence.*

**Response:** We have removed “For bats” from the sentence (lines 59-60).

*Lines 55-56: Inconsistent “to” vs “and” when demarcating a range of values. Use one style.*

**Response:** We have changed “between 140,000 to 679,000” changed to “between 140,000 and 679,000” (line 52).

*Lines 59-62: I think there are many scientists that would disagree with this statement that minimizes the effects of turbines. While it might not have an effect at the scale of overall bird mortality, there are likely key species where it does have a large effect, partly because of site location. The overall effects are likely to rise with increasing densities of wind farms at continental scales.*

**Response:** We agree that though populations of most bird species may not be affected significantly by collisions, some certainly are. We have modified the sentence to: “However, for most bird species, wind turbines contribute a small amount to total anthropogenic mortality [21] and may not be high enough to impact vital rates (e.g., annual survival probability) and state variables (e.g., abundance, density, occupancy) at the population level” (lines 55-58).

*Line 130: It’s unclear what the censoring of non-migratory species means. Are the authors simply reporting that they did not include non-migratory species in the analysis? Censor typically means one removed datapoints from a particular end of a distribution, hence my confusion.*

**Response:** Another reviewer also mentioned this. We meant to communicate that we removed data from the analysis and throughout the paper “censored” is replaced with “removed.”

*Line 148: The authors should cite Ameijeiras-Alonso et al. 2019 after the reference to ACR.
Ameijeiras-Alonso, J., Crujeiras, R.M. & Rodríguez-Casal, A. 2019. Mode testing, critical bandwidth and excess mass. TEST 28, 900–919.*

**Response:** We have moved the citation from the end of sentence to directly after mention of “ACR” (line 177).

*Line 149: It’s unclear to me how distances can be 0 if this represents the fall distance.*

**Response:** Distances of 0 represent carcasses that were found directly next to a turbine base. We have modified the sentence: “We set the lower limits for these tests at 0 m (carcasses with a distance of 0 m were found directly next to the turbine base) to reflect the non-negative nature of distance values” (lines 179-181)

*Line 150: Insert “to” so it reads “due to the right-skewness”.*

**Response:** We have revised this paragraph (lines 175-191), and this clause is no longer mentioned.

*Line 151: Not clear what “meaningful calculation of modes beyond this value” means. Are the authors implying that they limited the maximum distance to 60 m? Earlier (line 127), however, the authors wrote that they limited fall distances to 100 m. Please clarify both the terminology and the distance issues.*

**Response:** There was insufficient information (i.e. mortality records) beyond this distance for the ACR method to meaningfully calculate modes. We found that over 60 m, the algorithm began honing in on and identifying small clusters of observations as modes, when common sense suggested there were too little data to generalize these results. We are not implying that carcasses beyond 60 m are not meaningfully tied to turbine collision, but merely attempting to provide an honest accounting of the limitations of this analysis technique. We have revised our methods to expand our explanation for limiting mode testing to below 60 m (lines 175-191).

*Line 152: Likewise, I don’t understand the statement “We ran all other function parameters at default values”. Is this something inherent in package multimode? If so, provide more explanation about what these default values are and how they are used.*

**Response:** We would direct readers to our added supplementary R code for further detail on function use. We did not specify or explain default values in the manuscript body as doing so would have been redundant to referenced R package documentation, required many additional lines of text, and would not have advanced understanding or interpretation of the results.

*Line 162: passerines were already introduced at line 52. Thus, the parenthetical (Passeriformes) should appear at first mention, not here.*

**Response:** We have removed the references to Passeriformes and included it at the first mention of passerines.

*Line 168: Remove “identified” since it is redundant.*

**Response:** We have removed “identified.”

*Line 169: I think the authors should use different terminology here to avoid confusion. Normally, “federally listed” means an endangered or threatened species. In this case, they simply mean that the species is among those covered by the MBTA or NMBC.*

**Response:** We have changed the language to use “protected” instead of “federally listed.” The sentence now reads: “Of the 128 species, 123 were protected under the Migratory Bird Treaty Act [62] and 103 were protected under the Neotropical Migratory Bird Conservation Act [63]” (lines 202-204).

*Line 171: Again, “identified” is unnecessary to include. You’ve already said at line 162 that you are excluding those that were not identified. And you did not provide such caveats in other parts of this paragraph when referring to percentage fatalities (insectivores, upland birds, etc).*

**Response:** We have removed “identified.”

*Line 176: And, again, no need for “identified”. If it were not identified, it could not be assigned to species.*

Response: We have removed “identified.”

*Line 193: Scientific name for hoary was already given at line 73. As before, “excluding unidentified” is implied when one is reporting by species. Please delete.*

**Response:** We have removed the scientific name for hoary bat and removed “excluding unidentified.”

*Line 197: Is this 25% of facilities that reported bat mortality? If so, please clarify.*

**Response:** That is correct. We have updated the sentence to reflect this: “…less than 25% of facilities that reported bat mortality” (line 230).

*Line 198: The ordering of Table 4 is not intuitive. The first and last rows are redundant. All bats are in Chiroptera so this seems unnecessary to list. Why not just list each species, followed by a row with Unidentified Bat? I suggest deleting “CHIROPTERA, Unknown, and Vespertilionidae”. This information adds nothing.*

**Response:** We have followed your suggestion and removed “*CHIROPTERA, Unknown, and Vespertilionidae*” and we have moved “Unidentified Bat” to the bottom (line 231).

*Line 211: As before, are the authors using “censored” in this context as a synonym for “removed”? And, just to clarify, were any birds with distances >100 m set to 100 m or 60 m or deleted? Ambiguous as worded.*

**Response:** We have replaced “censored” with “removed” throughout the paper. We removed these records from the analysis entirely. We modified the sentence to reflect this: “We removed from our analyses nine bird fatality records with fall distances greater than 100 m, and 136 bird fatality records and 9 bat fatality records lacking species classification” (lines 243-246).

*Line 213: The authors use the word “removed” in this context and this is much clearer to the reader.*

**Response:** We have replaced “censored” with “removed” throughout the paper.

*Line 232: Bats should be plural in the figure caption. Please indicate what the error bars in panel A represent.*

**Response:** We changed “bat” to “bats.”

*Line 241: Please indicate what the error bars in panel A represent.*

**Response:** We have removed these figures. Please see our response to a similar comment regarding figure interpretation, Reviewer 1 Specific Comment Lines 232-237.

*Line 243: Typo in top-performing.*

**Response:** We changed “top-preforming” to “top-performing”

*Line 286: Too low to significantly impact total populations, given the current density and spatial extent of existing turbines. If the number of facilities increases, this effect may increase.*

**Response:** Yes, this impact may change as the number of wind facilities increases. We have added the following sentence to the third paragraph of our discussion section titled “Taxonomic and Temporal Patterns”: “At 25 or less fatalities per million individuals, even these are likely too low to significantly impact total population trajectories, but this effect may intensify as the number wind facilities increases” (lines 332-334).

*Line 289: Please provide a citation for the statement that “most populations are either stable or increasing”.*

**Response:** This reference was included in the table which contains this data. We have included a reference to that table (3; lines 209-210) in this sentence.

*Line 298: Need to add a hyphen to the compound modifier “population-level impacts”.*

**Response:** We have added a hyphen.

*Line 306: Please provide a citation for the Sep and May peaks in spring and fall migration activity.*

**Response:** We have added a reference and additionally modified the sentence to indicate that these peaks “presumably” correspond to migration activity (line 354).

*Line 309: Just to clarify, does “multiple mortality” mean that two individuals of the same species were found under the same turbine? Or at the same facility? Unclear terminology.*

**Response:** For bats, we defined multiple mortality events as greater than 10 bats at a given locality within a few days [71]. We have added this definition to our discussion (lines 356-359).
