# Supplementary material for: An evaluation of bird and bat mortality at wind turbines in the Northeastern United States
Source: PLoS One. 2020 Aug 28;15(8):e0238034. doi: 10.1371/journal.pone.0238034 (PMC7454995; doi:10.1371/journal.pone.0238034)
Supplement: Attachment — Submitted filename: Response to reviewers.docx [file pone.0238034.s028.docx]

Dear Dr. Magar,

My coauthors and I are grateful for the additional feedback provided by the reviewers, which has helped sharpen our manuscript. Enclosed is our second revision of our manuscript titled “An evaluation of bird and bat mortality at wind turbines in the Northeastern United States” (PONE-D-20-07129R1) that we wish to have considered for resubmission as an original research article in PLOS ONE. We believe that we have addressed the comments and concerns put forth by the reviewers, specifically those of Reviewer #3 related to our statistical analysis. For example, we have removed models with uninformative parameters. For some comments, we did not make changed in the manuscript but responded below, which we felt was appropriate. We have also added a few sentences to our discussion addressing points raised by Reviewer #3. Below, we respond to each reviewer’s comments individually. We have kept the original line numbers associated with each comment but in our response have included line numbers that reference the updated manuscript.

This resubmission represents original work that is not under consideration for publication elsewhere. Data in this manuscript are not included in previous publications nor will they be submitted for publication elsewhere. We are willing to cover page chargers and any other costs associated with the publication of the manuscript. This paper aims to make a contribution to the fields of conservation science, ornithology, mammalogy, and animal behavior, by reporting on patterns associated with bird and bat mortality at wind facilities, with an emphasis on the spatial arrangement of carcasses. To our knowledge, our dataset represents the largest ever compiled for bird and bat mortality at wind facilities in the Northeastern United States, an area where wind energy production is rapidly increasing. In addition, our empirical support for bird collisions with turbines poles in addition to blades is novel and germane for scientists, managers, and those involved in the wind power industry.

Thank you for your consideration.

Sincerely,

Daniel Choi

**Reviewer #1 General Comments**

General Comment 1

The authors have done a thorough job of addressing my prior comments and I believe the manuscript is now basically ready for publication. I have only a few minor specific suggestions:

**Reviewer #1 Specific Comments**

*Line 15:* I suggest replacing “benefits to humans” with “environmental benefits” to help keep the manuscript focused and because I imagine there are some people who would disagree with this statement.

**Response:** We have made this change, and the sentence now reads “Wind energy offers substantial environmental benefits, but wind facilities can negatively impact wildlife, including birds and bats” (line 15). We had added “to humans” in response to a suggestion by Reviewer #3 during the first round of revisions. They suggested that we needed to specify benefits to humans, as opposed to wildlife. We hope that specifying “environmental benefits” finds the middle ground.

*Line 59:* I suggest replacing “Wind development” with something more clear and specific such as “Wind-energy development”.

**Response:** We have made this change and the sentence now reads “Wind energy development may have greater implications for the conservation of bats” (line 60). We did not use the hyphen, as others have not in this context. For example, see references 8, 9, 20, 26, 74, and 88.

*Line 64:* I suggest inserting “individuals” after “949,000”.

**Response:** Thank you for your suggestion. However, we have not made this change, because we specify earlier in the sentence that the numbers describe annual fatalities, and we think this is clear. The sentence reads “Estimate of annual bat fatalities in North America have ranged between 600,000 and 949,000, but similar to birds, the number of US turbines has increased substantially since these estimates were developed [14]” (lines 65-67).

**Reviewer #2 General Comments**

General Comment 1

I have now read the revised manuscript by Choi et al. and find it to be MUCH improved from the first iteration. I appreciate the author's very careful consideration of all reviewers. The introduction reads very clearly and I believe the new analysis approach clears up the results and they are easier for the reader to understand.

**Response:** Thank you for compliment and feedback.

**Reviewer #2 Specific Comments**

*Line 170:* Minor comment on Line 170: I think you mean < 6.

**Response:** Thank you for catching this error. We have changed “> 6” to “< 6”.

**Reviewer #3 General Comments**

General Comment 1

I thank the authors for submitting a much-improved manuscript. It is always encouraging when authors respond so diligently to reviewer suggestions / comments. In particular, I am glad to see that the stepwise analysis has been removed. That was obviously a deal breaker for me. I am satisfied with all of their responses to my edits / comments / queries. I do have some issues related to the revised manuscript, as follows:

Statistical Issues

1. *Line 162:* I believe Harrison’s method of looking at dispersion parameter > 1 only pertains to GLMs. The authors are using GLMMs so I would expect this to be the ratio of the sum of the squared Pearson residuals to the residual degrees of freedom, as outlined in Harrison (2014).

**Response:** Thank you identifying this issue. We have updated our calculation of the dispersion parameter using the correct ratio (lines 166-167).

2. *Line 167:* Many information theorists will cringe at the “all combinations” approach of constructing candidate models. Dochtermann and Jenkins provide some good commentary on why one should not dredge candidate models. Dochtermann, N. & Jenkins, S. (2011) Developing multiple hypotheses in behavioral ecology. Behavioral Ecology and Sociobiology, 65, 37-45.

**Response:** We appreciate you raising this concern, and we agree that an “all combinations” approach is generally best avoided. In constructing our global models, we made conscious effort to avoid including terms that had no basis in theory. For this reason, we omitted interactions such as mass:turbine height where we could not conceive of meaningful relationships between the variables. We think that all terms included in our global models, and subsequently entered into the MuMIn package’s dredge function, represent informed hypotheses about the potential relationships between turbine or species characteristic and fall distance. Our use of “all combinations” refers to all combinations of terms from the global models, which are limited to what we expected to be informative parameters, not “all combinations” of a large set of variables and their interactions, as cautioned against by Dotchermann and Jenkins (2011). Unfortunately, despite the connotations they may bring to some readers, we must use the phrase “all combinations” and reference the “dredge” function to represent our methodology accurately.

3. How confident are the authors in using package multimode? Has this package been vetted at all? The citation they give (reference 58) is to a preprint repository.

**Response:** The preprint status of this manuscript also gave us pause. However, the multimode package has recently been cited in articles published in International Statistical Review, Electronic Journal of Statistics, Animal Migration, Fungal Ecology, Journal of the Acoustical Society of America, and GigaScience. The package’s documentation is extremely thorough not only on methodology, but also on theory. Additionally, we found that the authors’ methodology builds upon established and well-cited statistical literature. Based on these facts, we are highly confident in the package.

4. Table 6 looks to me like it includes uninformative parameters. For example, the top model AM and the 4th model AHM have identical log likelihoods. In this case, H is likely an uninformative parameter. You can see this again when you compare models A vs AH (they differ by one parameter but the log likelihoods are identical). Again, H is an uninformative parameter. See also AD vs ADH. Please refer to Figure 1 in Leroux, S.J. (2019) On the prevalence of uninformative parameters in statistical models applying model selection in applied ecology. PLoS ONE, 14, e0206711. Please check all of your AIC tables for such uninformative parameters. I also see some in Table 7, and maybe in Table 8 (although I’m not totally sure because of the interaction terms).

**Response** Thank you for the suggestion. In following this guidance, we identified several uninformative parameters in our model sets. We have added screening for uninformative parameters to our methodology (lines 173-175) and updated our results (line 262, tables 6-9) accordingly. Furthermore, we have included a helper function in our supplementary R code that operates on MuMIn package model average objects to automate a significant portion of the screening process. We hope that this code may encourage future implementation of Leroux’s recommendations. We have kept models with uninformative variables in the tables following Arnold, T.W. (2010), Uninformative Parameters and Model Selection Using Akaike's Information Criterion. The Journal of Wildlife Management, 74: 1175-1178. doi:[10.1111/j.1937-2817.2010.tb01236.x](https://doi.org/10.1111/j.1937-2817.2010.tb01236.x)

Reviewer #3 Specific Comments

*Line 21:* Like you do at 77, you should define “fall distance” here in the Abstract.

**Response:** We have built this definition into the preceding sentence, which now reads “Understanding the horizontal fall distance between a carcass and the nearest turbine pole is important in designing effective search protocols and estimating total mortality” (lines 19-20).

*Line 25:* Compound modifiers “long-distance migratory”, “short-distance migrants” need hyphens;

**Response:** Thank you for pointing this out. We have added hyphens to this terms throughout.

*Line 44:* Extra period at end of sentence.

**Response:** We have removed the extra period.

*Line 46:* United States is abbreviated to US here but not in the Abstract at 21 and 22, where only the abbreviation is used without first defining it.

**Response:** We added “United States” to the sentence in the abstract, which now reads “We explored patterns in taxonomic composition and fall distance of bird and bat carcasses at wind facilities in the Northeastern United States using publicly available data and data submitted to the US Fish and Wildlife Service under scientific collecting and special purpose utility permits for collection and study of migratory birds” (lines 20-24). We additionally removed the use of the abbreviation “US” and “USFWS” in the abstract and instead define these upon first mention in the main body at lines 47 and 104. We did not change “US” in the title “US Fish and Wildlife Service,” as this is part of the official name of the agency.

*Line 62:* Suggest rewording “At the majority of US wind facilities that have been examined, bat mortality is estimated to be higher than bird mortality”.

**Response:** We made this change with small revisions: “At the majority of US wind facilities that have been examined, bat mortality has been estimated to be higher than bird mortality [2, 6]” (lines 63-65).

*Line 95:* Suggest replacing “unstandardized” with “non-standardized”.

**Response:** We have made this change.

*Line 115:* Sentence is framed in past tense so it should be “allowed”

**Response:** We have made this change.

*Line 145:* Insert “to” so it reads “we assigned records to the most…”

**Response:** We have inserted “to” before “records”, instead of after, as this would have changed the meaning. Added “each.” The sentence now reads “Where turbine dimensions varied within an individual facility, we assigned to each record the most common hub height and rotor diameter for that facility” (lines 145-147).

*Line 160:* It’s not clear to me why there would be more than one global model? Or are the authors referring to the birds-only, bats-only, both global models?

**Response:** We are referring to the global models and have inserted a parenthetical statement clarifying this: “Next, we measured the fit of each global model (birds-only, bats-only, and all-taxa global models) by using the MuMIn package [52] in R to calculate a marginal and conditional pseudo r-squared based on the Trigamma-estimate method” (lines 161-164).

*Line 199:* “foliage-gleaning insectivores” missing hyphen

**Response:** We have inserted a hyphen.

*Line 215:* turkey vulture taxonomic name has not been provided.

**Response:** We have added the taxonomic name for turkey vulture.

*Line 225:* hoary needs taxonomic name

**Response:** We have added the taxonomic name for hoary bat.

*Line 247:* Check the information for Reference 48. It is no longer referred to as The Birds of North America.

**Response:** Thank you for pointing this out. We have changed the reference here and in the references section.

*Line 289:* Insert hyphen “short-distance migrants”

**Response:** We have inserted a hyphen.

*Line 290:* Ditto “long-distance migrants”

**Response:** We have inserted a hyphen.

*Line 325:* Unclear what “Species were also comparable” means. Comparable to other studies?

**Response:** We have added “to other studies.” The sentence now reads “Species were also comparable to other studies, predominantly red-eyed vireo, golden-crowned kinglet, and magnolia warbler” (lines 332-333).

*Line 327:* “in the east” is much too casual. I assume you mean the eastern US?

**Response:** We have added “ern US.” The sentence now reads “Raptors made up just 5% of fatalities, similar to other reports in the eastern US [34], but lower than raptor mortality along the west coast of the US [34, 66]” (lines 334-335).

*Line 328:* Again, spell it out. “west coast of the US”.

**Response:** We have specified the west coast, and the sentence now reads “Raptors made up just 5% of fatalities, similar to other reports in the eastern US [34], but lower than raptor mortality along the west coast of the US [34, 66]” (lines 334-335).

*Line 333:* Insert “of” so it reads “number of wind facilities”

**Response:** We have inserted “of”.

*Line 334:* Probably better to write “over the past 50 years”

**Response:** We have changed this to “over the past 50 years.”

*Line 337:* Use past tense “carcasses were recovered”

**Response:** We have changed to a past tense.

*Line 342:* Unclear what the 90% refers to? That these three species made up 90% of bat mortalities?

**Response:** We have added clarification in a parenthetical and the sentence now reads “Three migratory tree bats (hoary bat, silver-haired bat, and eastern red bat) were somewhat more common (90% of all bat fatalities in our data) than reported elsewhere [22, 68], but our data represent a slightly different geographic extent” (lines 349-351).

*Line 357:* Or biased reporting? What the authors more generously refer to as “reporting culture” on L399.

**Response:** Thank you for pointing this out. We have added “biased reporting” to this sentence, which now reads “This may be due to limited sample size, geographic differences, or biased reporting” (lines 365-366).

*Line 369:* Ambiguous. Differ from the present study or differ between large and small birds?

**Response:** We have clarified this sentence, which now reads: “Moreover, while AWWI’s [34] distance distributions did not appear to differ between large and small birds, this may have been the result of overly broad size bins” (lines 376-377).

*Line 380:* Seems odd to put the references in the middle of the sentence, given that the main message is that turbine height and blade length relate to fall distance. Suggest putting the reference at the end.

**Response:** Thank you for pointing this out. We have moved the reference to the end of the sentence.

*Lines 381-383:* this seems redundant with 370-373.

**Response:** Thank you for commenting on this. These sentences represent two different scenarios. With small turbines, larger birds are likely to fall farther from turbines. Similarly, *but independently*, larger turbines will have larger fall distances associated with them for all birds, regardless of size. Hence, we provide two different rationale for considering expansion of search areas/zones. As such, we feel the verbiage should remain as is.

*Line 391:* I also think the authors should comment on how they assigned one value to all turbines at a facility. The fact that facility ID accounts for such high variation is important as it relates to variability in spatial arrangement and heights / designs of turbines at the facility. There could be particular areas of the facility that have higher mortality but that information is lost because the authors were not given turbine-specific mortality data. This is an important point and may show as much variation within a facility as there is among facilities. For example, topography within some facilities is likely to vary a lot whereas others may be more uniform in such factors.

**Response:** Thank you for suggesting this. We have added three additional sentences. First, we comment on the spatial arrangement of turbines within a facility: “For example, spatial patterns may be different at wind facilities with turbines arranged in a line (e.g., along a mountain ridge) than at those with turbines more evenly spaced in a group (e.g., in agricultural fields)” (lines 405-407). Second, we comment on how we used just one set of turbine dimensions per facility: “Variation between individual turbines within a wind facility may also affect spatial patterns of fall distance. For a few wind facilities which contained turbines of different sizes, we used only the most common turbine dimensions in our analysis, and this may have contributed to the variation accounted for by the random effect of facility ID” (lines 410-413).

*Line 413:* It might be better to use “taxon-specific” rather than “taxa-specific”

**Response:** We have made this change.

*Lines 452-462:* This paragraph seems redundant with the previous paragraph. In particular, see 441-443.

**Response:** Thank you for your comment. We think that these paragraphs are disparate enough to warrant inclusion of each. The first paragraph points to specific findings of our study and how the observed phenomenon could result in over or underestimation of mortality. We have modified the verbiage of the first paragraph in an effort to make this more clear (lines 455-459). The second paragraph is focused on the greater implications of collisions of birds with wind turbines poles.

*Line 467:* What is a “curtailment regime”?

**Response:** We have inserted a parenthetical definition, and the sentence now reads “Additionally, the distance distribution we present is based on various mortality survey procedures, surveyor expertise, curtailment regimes (purposeful reduction in turbine operation and electricity generation), and turbine models and sizes” (lines 480-483).

*Lines 473-475:* Not to mention weather patterns.

**Response:** Thank you for pointing this out. We have included mention of different weather patterns alongside our mention of geographic areas. The sentence now reads “For example, almost no turbines along the coast of the Great Lakes and Massachusetts are included in this dataset, and trends may be different for shorebirds or seabirds in certain geographic areas and areas with different weather patterns” (lines 488-490).

*Line 501:* Suggest rewording as “assisted with data collection”

**Response:** We have made this change.

*Line 505:* Reference seems incomplete. No place of publication, etc.

**Response:** Thank you for your attention to detail and for catching these many errors in our literature cited section. For this reference, no place of publication, other than the “United States,” is available, but we have added this. Further, no other information is available, but we have changed the standard link to a DOI link.

*Line 559:* References in Annual Reviews do not typically indicate editors. They are more typically cited like journal articles.

**Response:** We have removed the editors from the citation.

*Line 561:* Reference is incomplete. No publisher, place of publication.

**Response:** We have added this information.

*Line 567:* Extra space on “wind-energy”

**Response:** We have removed the extra space.

*Line 584:* Journal name is not abbreviated or capitalized.

**Response:** We have abbreviated and capitalized the journal name.

*Line 592:* Is it correct that the paper is only one page?

**Response:** We have filled in the correct page numbers.

*Line 593:* Incomplete reference.

**Response:** We have added the necessary information.

*Line 600:* Check reference. Journal name not abbreviated, only one page listed.

**Response:** We have added the correct page numbers. However, this is not a journal and does not have an accepted abbreviation.

*Line 618:* Check style on title – every word is capitalized.

**Response:** We have changed title to lowercase.

*Line 629:* Reference is out of date. It is no longer referred to as Birds of North America.

**Response:** We have updated the reference.

*Line 646:* Extra space on “information-theoretic”

**Response:** We have removed the extra space.

*Line 661:* Incomplete reference.

**Response:** We have updated this reference as per official PLOS ONE guidelines <https://www.nlm.nih.gov/bsd/uniform_requirements.html>

*Line 663:* Incomplete reference.

**Response:** We have updated this reference as per official PLOS ONE guidelines <https://www.nlm.nih.gov/bsd/uniform_requirements.html>

*Line 667:* Incomplete reference. No place of publication.

**Response:** We have added this information.

*Line 669:* Ditto to above.

**Response:** We have added this information.

*Line 673:* Ditto to above.

**Response:** We have added this information.

*Line 682:* Italicize genera.

**Response:** We have made this change.

*Line 706:* Is this a journal? One page? Please check.

**Response:** We have updated this with the correct reference from the Journal, *Wind Energy Science*.

*Line 734:* Incomplete reference.

**Response:** We have updated this reference.

*Line 741:* Incomplete reference.

**Response:** We have updated this reference.

*Line 744:* Incomplete reference.

**Response:** We have updated this reference.

*Line 747:* Incomplete reference.

**Response:** We have updated this reference.

*Line 758:* Please check figure S2. When I downloaded it, it was blank.

**Response:** We have re-uploaded this figure.

*Line 833:* The R code annotation is beautiful! Kudos to the second author.

**Response:** Thank you for your compliment.
