# Supplementary material for: An evaluation of bird and bat mortality at wind turbines in the Northeastern United States
Source: PLoS One. 2020 Aug 28;15(8):e0238034. doi: 10.1371/journal.pone.0238034 (PMC7454995; doi:10.1371/journal.pone.0238034)
Supplement: Attachment — Submitted filename: Response to reviewers.docx [file pone.0238034.s029.docx]

Dear Dr. Magar,

My coauthors and I are grateful for the additional feedback provided by the reviewers, which has helped sharpen our manuscript. Enclosed is our third revision of our manuscript titled “An evaluation of bird and bat mortality at wind turbines in the Northeastern United States” (PONE-D-20-07129R2) that we wish to have considered for resubmission as an original research article in PLOS ONE. We believe that we have addressed the brief comments put forth by Reviewer #3. We have also added two minor sentences to our discussion (lines 451-454) to mention a finding by the recent paper by Stokke et al. 2020, “Effect of tower base painting on willow ptarmigan collision rates with wind turbines,” which provides evidence for pole collisions by willow ptarmigan (*Lagopus lagopus*). Though this addition does not alter our discussion points in any way, we felt its inclusion was merited in our discussion of pole collisions and provided an important reference. Below, we respond to each reviewer’s comments individually. We have kept the original line numbers associated with each comment but in our response have included line numbers that reference the updated manuscript.

This resubmission represents original work that is not under consideration for publication elsewhere. Data in this manuscript are not included in previous publications nor will they be submitted for publication elsewhere. We are willing to cover page chargers and any other costs associated with the publication of the manuscript. This paper aims to make a contribution to the fields of conservation science, ornithology, mammalogy, and animal behavior, by reporting on patterns associated with bird and bat mortality at wind facilities, with an emphasis on the spatial arrangement of carcasses. To our knowledge, our dataset represents the largest ever compiled for bird and bat mortality at wind facilities in the Northeastern United States, an area where wind energy production is rapidly increasing. In addition, our empirical support for bird collisions with turbines poles in addition to blades is novel and germane for scientists, managers, and those involved in the wind power industry.

Thank you for your consideration.

Sincerely,

Daniel Choi

**Reviewer #3 Comments**

General Comment 1

*As* before, the authors have done a great job at addressing my concerns and suggested edits. This manuscript will be an important contribution to the literature. It’s been a pleasure working with such attentive authors.

**Response:** We thank you for your continual investment in this paper and for reviewing each draft in such a thorough and timely manner.

I have a few trivial line edits:

*Line 158:* You are not really fitting models, you are ranking them.

**Response:** We have replaced “fitting” with “ranking.” The sentence now reads “Ranking models according to the Information-Theoretic approach [49], we first constructed global models containing all applicable predictor variables (Table 1)” (lines 158-159).

*Line 174:* Typo “be” should be “by”

**Response:** We have made this change.

*Line 196:* I think you should use “0”, not “zero” in this context.

**Response:** We have made this change.

*Table 3:* I think it would be better to just report Total Population in millions. The extra zeros are clumsy.

**Response:** Thank you for pointing this out. We have made this change. For example, instead of “130,000,000”, population numbers are listed as “130 million”. Instead of listing the column header as “Total Population (millions)”, we decided to include “million” after each value in the table, as we thought this created more ease for the reader.

*Line 411:* Incorrect use of a restrictive clause. Change “which” to “that.

**Response:** We have made this change.

*Line 447:* Extra period.

**Response:** We have removed the extra period.
